# Supplementary material for: Spatio-temporal dynamics of three diseases caused by Aedes-borne arboviruses in Mexico
Source: Commun Med (Lond). 2022 Oct 28;2:134. doi: 10.1038/s43856-022-00192-7 (PMC9616936; doi:10.1038/s43856-022-00192-7)
Supplement: Supplementary file 1 — Supplementary Information [file 43856_2022_192_MOESM1_ESM.pdf]

Supplement text

Spatio-temporal dynamics of three diseases caused by *Aedes*-borne arboviruses in Mexico

Bo Dong[1], Latifur Khan[1], Madison Smith[2], Jesus Trevino[3], Bingxin Zhao[4], Gabriel L. Hamer[5], Alex Lemus[6], Aracely Angulo Molina[7], Jailos Lubinda[8], Uyen-Sa DT Nguyen[2], Ubydul Haque[2]

*Corresponding author. Email: mdubydul.haque@unthsc.edu

Supplementary Notes 1

KNN: Neighbours-based classification is a type of lazy learning as it does not attempt to construct a general internal model, but simply stores instances of the training data. Classification is computed from a simple majority vote of the k nearest neighbors of each point.

Advantages: This algorithm is simple to implement, robust to noisy training data, and effective if training data is large.

Disadvantages: Need to determine the value of K and the computation cost is high as it needs to compute the distance of each instance to all the training samples.

Decision Tree: Given a data of attributes together with its classes, a decision tree produces a sequence of rules that can be used to classify the data.

Advantages: The decision Tree is simple to understand and visualize, requires little data preparation, and can handle both numerical and categorical data.

Disadvantages: Decision trees can create complex trees that do not generalize well, and decision trees can be unstable because small variations in the data might result in a completely different tree being generated.

Random Forest: Random forest classifier is a meta-estimator that fits a number of decision trees on various sub-samples of datasets and uses an average to improve the predictive accuracy of the model and controls over-fitting. The sub-sample size is always the same as the original input sample size but the samples are drawn with replacement.

Advantages: Reduction in over-fitting and random forest classifiers is more accurate than decision trees in most cases.

Disadvantages: Slow real-time prediction, difficult to implement, and complex algorithm.

SVM: Support vector machine is a representation of the training data as points in space separated into categories by a clear gap that is as wide as possible. New examples are then mapped into that same space and predicted to belong to a category based on which side of the gap they fall.

Advantages: Effective in high dimensional spaces and uses a subset of training points in the decision function so it is also memory efficient.

Disadvantages: The algorithm does not directly provide probability estimates, these are calculated using an expensive five-fold cross-validation.

Neural network: is based on a collection of connected units or nodes called artificial neurons, which loosely model the neurons in a biological brain.

Advantages: can simulate complex functions includes linear and non-linear functions.

Disadvantages: need a lot of time for training. Need a lot of time for tuning parameters.

XGBoost stands for Extreme Gradient Boosting; it is a specific implementation of the Gradient Boosting method which uses more accurate approximations to find the best tree model. It employs a number of nifty tricks that make it exceptionally successful, particularly with structured data. The most important is computing second-order gradients, i.e. second partial derivatives of the loss function (similar to Newton's method), which provides more information about the direction of gradients and how to get to the minimum of our loss function. While regular gradient boosting uses the loss function of our base model (e.g. decision tree) as a proxy for minimizing the error of the overall model, XGBoost uses the 2nd order derivative as an approximation.

Supplementary Discussion

We simultaneously ran a different algorithm based on clustering information to show the influence of socio-economic status (SES) attributes and climate attributes for all clusters and non-clusters.

We sampled the number of CHIKV, DENV, and ZIKV from the whole data based on this location list. Furthermore, we ran the different algorithms to show the influence of socioeconomics and the climate attributes for all clusters.

Socio-economic features have around 38.97% more impact than climatic features on model output in cluster 1 (SHAP value). Climate features have around 10.12% more impact than socio-economic features on model output in cluster 1 (RDC). Climate features (Pearson) have around 0.8% more impact than socio-economic features on model output in cluster 1 (figure S2).

Socio-economic features have around 37.23% more impact than climatic features on model output in cluster 2. Socio-economic features have around 5.56% more impact than climate features on model output in cluster 2. Socio-economic features have around 26.20% more impact than climate features on model output in cluster 2 (figure S4).

Socio-economic features have around 28.02% more impact than climatic features on model output in cluster 3. Climate features have around 16.72% more impact than socio-economic features on the model output in cluster 3. Climate features have around 4.38% more impact than socio-economic features on model output in cluster 3 (figure S5).

Climatic features have around 9.32% more impact than socio-economic features on model output in cluster 4. Climate features have around 14.40% more impact than socio-economic features on model output in cluster 4. Socio-economic features have around 10.00% more impact than climate features on model output in cluster 4.

Socio-economic features have around 26.84% more impact than climatic features on model output in cluster 5. Climate features have around 23.66% more impact than socio-economic features on model output in cluster 5. Climatic features have around 29.76% more impact than socio-economic features on model output in cluster 5.

Socio-economic features have around 23.60% more impact than climatic features on model output in cluster 6. Climate features have around 12.76% more impact than socio-economic

features on model output in cluster 6. Climatic features have around 2.33% more impact than socio-economic features on model output in cluster 6.

Socio-economic features have around 0.84% more impact than climatic features on model output in cluster 7. Climate features have around 7.66% more impact than socio-economic features on model output in cluster 7. Climatic features have around 4.78% more impact than socio-economic features on model output in cluster 7.

Socio-economic features have around 47.21% more impact than climatic features on model output in cluster 8. Climate features have around 11.14% more impact than socio-economic features on model output in cluster 8. Socio-economic features have around 6.84% more impact than climatic features on model output in cluster 8.

Socio-economic features have around 43.96% more impact than climatic features on model output in cluster 9. Socio-economic features have around 23.90% more impact than climate features on model output in cluster 9. Socio-economic features have around 38.51% more impact than climatic features on model output in cluster 9.

Socio-economic features have around 35.58% more impact than climatic features on model output in cluster 10. Socio-economic features have around 6.60% more impact than climate features on model output in cluster 10. Socio-economic features have around 34.96% more impact than climatic features on model output in cluster 10.

Socio-economic features have around 17.58% more impact than climatic features on model output in cluster 11. Climate features have around 1.14% more impact than socio-economic features on model output in cluster 11. Socio-economic features have around 20.83% more impact than climatic features on model output in cluster 11.

Climatic features have around 23.44% more impact than socio-economic features on model output in cluster 12. Climate features have around 8.92% more impact than socio-economic features on model output in cluster 12. Climatic features have around 17.63% more impact than socio-economic features on model output in cluster 12.

Table S2-S13 shows the prediction results of different machine learning approaches for DENV clusters. Table S14 shows the weights of socio-economic features and climate features by using SHAP, RDC, and Pearson approaches. Prediction results of different machine learning approaches for ZIKV clusters are presented in tables S15 - S20. Table S21 shows the weights of socio-economic features and climate features by using SHAP, RDC, and Pearson approaches. Prediction results of different machine learning approaches for CHIKV clusters are presented in Table S22 - 24. The weights of socio-economic features and climate features by using SHAP, RDC, and Pearson approaches are presented in Table S25.

Supplementary figure legends

Figure S1: Spatial distribution of rainfall and temperature in Mexico

Figure S2: Socio-economic zones in Mexico

Figure S3: CHIKV cluster 1

Figure S4: CHIKV cluster 2

Figure S5: CHIKV cluster 3

Figure S6: DENV cluster 1

Figure S7: DENV cluster 2

Figure S8: DENV cluster 3

Figure S9: DENV cluster 4

Figure S10: DENV cluster 5

Figure S11: DENV cluster 6

Figure S12: DENV cluster 7

Figure S13: DENV cluster 8

Figure S14: DENV cluster 9

Figure S15: DENV cluster 10

Figure S16: DENV cluster 11

Figure S17: DENV cluster 12

Figure S18: ZIKV cluster 1

Figure S19: ZIKV cluster 2

Figure S20: ZIKV cluster 3

Figure S21: ZIKV cluster 4

Figure S22: ZIKV cluster 5

Figure S23: ZIKV cluster 6

Figure S24: Average confirmed cases for CHIKV, DENV, and ZIKV from Jan to Dec over 2012-2019

Supplementary Tables

Table S1: Socio-economic and climate features impact on DENV, ZIKV and CHIKV virus prediction at different clusters

	DENV				ZIKV				CHIKV			
	Majority vote		Average		Majority vote		Average		Majority vote		Average	
	socio-economic	Climate	socio-economic	Climate	socio-economic	Climate	socio-economic	Climate	socio-economic	Climate	socio-economic	Climate
Cluster1	0.47	0.53	0.47	0.53	0.33	0.67	0.42	0.58	0.43	0.57	0.46	0.54
Cluster2	0.63	0.37	0.63	0.37	0.44	0.56	0.46	0.54	0.43	0.57	0.43	0.57
Cluster3	0.45	0.55	0.52	0.48	0.44	0.56	0.50	0.50	0.31	0.69	0.41	0.59
Cluster4	0.44	0.56	0.40	0.60	0.63	0.37	0.51	0.49				
Cluster5	0.36	0.64	0.41	0.59	0.59	0.41	0.49	0.51				
Cluster6	0.45	0.55	0.47	0.53	0.59	0.41	0.51	0.49				
Cluster7	0.44	0.56	0.44	0.56								
Cluster8	0.56	0.44	0.52	0.48								
Cluster9	0.65	0.35	0.56	0.44								
Cluster10	0.60	0.40	0.51	0.49								
Cluster11	0.65	0.35	0.60	0.40								
Cluster12	0.41	0.59	0.41	0.59								
All clusters average	0.51	0.49	0.50	0.50	0.51	0.49	0.48	0.52	0.53	0.47	0.43	0.57

Dengue:

For cluster 1:

Table S2: Prediction results of different machine learning approaches for cluster 1 in Dengue clusters

Method	Accuracy	Weighted accuracy	Precision	Recall	F-score
XGBoost	0.8895	0.7254	0.8283	0.7254	0.7651
Decision tree	0.8543	0.7343	0.7323	0.7343	0.7342
SVM	0.8513	0.5357	0.7754	0.5357	0.5328
KNN	0.8649	0.7132	0.7436	0.7132	0.7253
Random forest	0.8862	0.7047	0.8188	0.7047	0.7456
Neural network	0.8911	0.7257	0.8180	0.7257	0.7595

For cluster 2:

Table S3: Prediction results of different machine learning approaches for cluster 2 in Dengue clusters

Method	Accuracy	Weighted accuracy	Precision	Recall	F-score
XGBoost	0.8923	0.8976	0.8998	0.8976	0.8963
Decision tree	0.8311	0.8284	0.8327	0.8284	0.8268
SVM	0.7753	0.7737	0.7769	0.7737	0.7786
KNN	0.8441	0.8357	0.8545	0.8357	0.8395
Random forest	0.8748	0.8658	0.8821	0.8658	0.8673
Neural network	0.7792	0.7757	0.7821	0.7757	0.7765

For cluster 3:

Table S4: Prediction results of different machine learning approaches for cluster 3 in Dengue clusters

Method	Accuracy	Weighted accuracy	Precision	Recall	F-score
XGBoost	0.9154	0.8189	0.8665	0.8189	0.8378
Decision tree	0.8911	0.7965	0.8087	0.7965	0.8032
SVM	0.8358	0.5	0.4182	0.5	0.4561
KNN	0.8936	0.7636	0.8123	0.7636	0.7834
Random forest	0.9115	0.7835	0.8758	0.7835	0.8169
Neural network	0.8875	0.6958	0.8576	0.6958	0.7445

For cluster 4:

Table S5: Prediction results of different machine learning approaches for cluster 4 in Dengue clusters

Method	Accuracy	Weighted accuracy	Precision	Recall	F-score
XGBoost	0.8784	0.7868	0.8232	0.7868	0.8046
Decision tree	0.8389	0.7568	0.7434	0.7568	0.7488
SVM	0.8223	0.5840	0.7880	0.5840	0.5967
KNN	0.8314	0.7259	0.7369	0.7259	0.7341
Random forest	0.8758	0.7545	0.8196	0.7545	0.7863
Neural network	0.8434	0.6789	0.7865	0.6789	0.7069

For cluster 5:

Table S6: Prediction results of different machine learning approaches for cluster 5 in Dengue clusters

Method	Accuracy	Weighted accuracy	Precision	Recall	F-score
XGBoost	0.9076	0.7731	0.8568	0.7731	0.8052
Decision tree	0.8724	0.7600	0.7611	0.7600	0.7605
SVM	0.8482	0.5468	0.7543	0.5468	0.5398
KNN	0.8679	0.6986	0.7567	0.6986	0.7215
Random forest	0.8948	0.7357	0.8435	0.7357	0.7743
Neural network	0.8826	0.6776	0.8247	0.6776	0.7165

For cluster 6:

Table S7: Prediction results of different machine learning approaches for cluster 6 in Dengue clusters

Method	Accuracy	Weighted accuracy	Precision	Recall	F-score
XGBoost	0.9698	0.9072	0.9026	0.9072	0.9048
Decision tree	0.9519	0.8617	0.8617	0.8617	0.8617
SVM	0.9198	0.6479	0.7765	0.6479	0.6867
KNN	0.9548	0.8838	0.8457	0.8838	0.8643
Random forest	0.9669	0.8760	0.9238	0.8760	0.8978
Neural network	0.9565	0.8059	0.9367	0.8059	0.8601

For cluster 7:

Table S8: Prediction results of different machine learning approaches for cluster 7 in Dengue clusters

Method	Accuracy	Weighted accuracy	Precision	Recall	F-score
XGBoost	0.8958	0.7649	0.8165	0.7649	0.7847
Decision tree	0.8654	0.7659	0.7557	0.7659	0.7635
SVM	0.8452	0.5347	0.7257	0.5347	0.5148
KNN	0.8680	0.7176	0.7435	0.7176	0.7258
Random forest	0.8917	0.7369	0.8278	0.7369	0.7775
Neural network	0.8762	0.6835	0.7975	0.6835	0.7189

For cluster 8:

Table S9: Prediction results of different machine learning approaches for cluster 8 in Dengue clusters

Method	Accuracy	Weighted accuracy	Precision	Recall	F-score
XGBoost	0.8454	0.8057	0.8024	0.8057	0.8040
Decision tree	0.8326	0.8047	0.7765	0.8047	0.7898
SVM	0.7402	0.5	0.3701	0.5	0.4253
KNN	0.8225	0.7534	0.7754	0.7534	0.7631
Random forest	0.8546	0.8058	0.8162	0.8058	0.8112
Neural network	0.7736	0.6286	0.7020	0.6286	0.6432

For cluster 9:

Table S10: Prediction results of different machine learning approaches for cluster 9 in Dengue clusters

Method	Accuracy	Weighted accuracy	Precision	Recall	F-score
XGBoost	0.7321	0.7146	0.7031	0.7146	0.7047
Decision tree	0.6851	0.6783	0.6578	0.6783	0.6626
SVM	0.6842	0.5	0.3421	0.5	0.4062
KNN	0.6848	0.5876	0.6149	0.5876	0.5937
Random forest	0.8947	0.8387	0.9346	0.8387	0.8659
Neural network	0.6876	0.5	0.3421	0.5	0.4062

For cluster 10:

Table S11: Prediction results of different machine learning approaches for cluster 10 in Dengue clusters

Method	Accuracy	Weighted accuracy	Precision	Recall	F-score
XGBoost	0.7769	0.7523	0.7460	0.7523	0.7489
Decision tree	0.7438	0.7156	0.7078	0.7156	0.7111
SVM	0.6724	0.5	0.3354	0.5	0.4016
KNN	0.8259	0.7638	0.8341	0.7638	0.7842
Random forest	0.8103	0.7669	0.7914	0.7669	0.7752
Neural network	0.6736	0.5	0.3378	0.5	0.4034

For cluster 11:

Table S12: Prediction results of different machine learning approaches for cluster 11 in Dengue clusters

Method	Accuracy	Weighted accuracy	Precision	Recall	F-score
XGBoost	0.8875	0.6567	0.7569	0.6567	0.6962
Decision tree	0.8518	0.6648	0.6621	0.6648	0.6646
SVM	0.8765	0.5	0.4389	0.5	0.4671
KNN	0.8814	0.6578	0.7273	0.6578	0.6831
Random forest	0.9045	0.6652	0.8316	0.6652	0.7128
Neural network	0.8740	0.5	0.4370	0.5	0.4664

For cluster 12:

Table S13: Prediction results of different machine learning approaches for cluster 12 in Dengue clusters

Method	Accuracy	Weighted accuracy	Precision	Recall	F-score
XGBoost	0.8258	0.7347	0.7768	0.7347	0.7521
Decision tree	0.7254	0.6775	0.6534	0.6775	0.6632
SVM	0.7478	0.5	0.3739	0.5	0.4278
KNN	0.8043	0.7034	0.7535	0.7034	0.7176
Random forest	0.8434	0.7239	0.8367	0.7239	0.7565
Neural network	0.7478	0.5	0.3739	0.5	0.4278

Table S14: weights of socio-economic features and climate features by using SHAP, RDC, and Pearson approaches.

	SHAP		RDC		Pearson	
	socio-economic	Climate	socio-economic	Climate	socio-economic	Climate
Cluster1	0.4840	0.5160	0.4494	0.5506	0.4959	0.5041
Cluster2	0.7388	0.2612	0.5278	0.4722	0.6310	0.3690
Cluster3	0.6880	0.3120	0.4164	0.5836	0.4781	0.5219
Cluster4	0.2381	0.7619	0.4280	0.5720	0.5500	0.4500
Cluster5	0.5116	0.4884	0.3817	0.6183	0.3511	0.6488
Cluster6	0.5028	0.4972	0.4362	0.5638	0.4883	0.5116
Cluster7	0.3947	0.6053	0.4617	0.5383	0.4761	0.5239
Cluster8	0.5941	0.4059	0.4443	0.5557	0.5342	0.4657
Cluster9	0.3948	0.6052	0.6195	0.3805	0.6925	0.3074
Cluster10	0.3226	0.6773	0.5330	0.4670	0.6748	0.3251
Cluster11	0.7105	0.2895	0.4943	0.5057	0.6041	0.3958
Cluster12	0.3651	0.6349	0.4554	0.5446	0.4118	0.5881

Zika:

For cluster 1:

Table S15: Prediction results of different machine learning approaches for cluster 1 in Zika clusters

Method	Accuracy	Weighted accuracy	Precision	Recall	F-score
XGBoost	0.9854	0.5345	0.7131	0.5345	0.5546
Decision tree	0.9792	0.5747	0.5654	0.5747	0.5721
SVM	0.9883	0.5	0.4941	0.5	0.4970
KNN	0.9866	0.5175	0.5777	0.5175	0.5271
Random forest	0.9883	0.5347	0.7445	0.5347	0.5532
Neural network	0.9866	0.5822	0.6682	0.5822	0.6098

For cluster 2:

Table S16: Prediction results of different machine learning approaches for cluster 2 in Zika clusters

Method	Accuracy	Weighted accuracy	Precision	Recall	F-score
XGBoost	0.9736	0.6289	0.7232	0.6289	0.6581
Decision tree	0.9677	0.5954	0.5889	0.5954	0.5921
SVM	0.9805	0.5	0.4902	0.5	0.4950
KNN	0.9729	0.5311	0.5575	0.5311	0.5396
Random forest	0.9828	0.5748	0.8232	0.5748	0.6127
Neural network	0.9812	0.5376	0.8242	0.5376	0.5568

For cluster 3:

Table S17: Prediction results of different machine learning approaches for cluster 3 in Zika clusters

Method	Accuracy	Weighted accuracy	Precision	Recall	F-score
XGBoost	0.9745	0.5636	0.7376	0.5636	0.5953
Decision tree	0.9646	0.6158	0.6378	0.6158	0.6268
SVM	0.9768	0.5	0.4884	0.5	0.4941
KNN	0.9754	0.5259	0.6564	0.5259	0.5472
Random forest	0.9743	0.5746	0.8035	0.5746	0.6178
Neural network	0.9768	0.5152	0.7387	0.5152	0.5235

For cluster 4:

Table S18: Prediction results of different machine learning approaches for cluster 4 in Zika clusters

Method	Accuracy	Weighted accuracy	Precision	Recall	F-score
XGBoost	0.9791	0.7446	0.7446	0.7446	0.7446
Decision tree	0.9791	0.7446	0.7446	0.7446	0.7446
SVM	0.9791	0.5	0.4895	0.5	0.4947
KNN	0.9895	0.75	0.9947	0.75	0.8306
Random forest	0.9895	0.75	0.9947	0.75	0.8306
Neural network	0.9895	0.75	0.9947	0.75	0.8306

For cluster 5:

Table S19: Prediction results of different machine learning approaches for cluster 5 in Zika clusters

Method	Accuracy	Weighted accuracy	Precision	Recall	F-score
XGBoost	0.9848	0.6246	0.6950	0.6246	0.6521
Decision tree	0.9821	0.6235	0.6354	0.6235	0.6276
SVM	0.9869	0.5	0.4934	0.5	0.4967
KNN	0.9837	0.5624	0.6178	0.5624	0.5787
Random forest	0.9837	0.5659	0.9956	0.5659	0.6082
Neural network	0.9854	0.5	0.4934	0.5	0.4967

For cluster 6:

Table S20: Prediction results of different machine learning approaches for cluster 6 in Zika clusters

Method	Accuracy	Weighted accuracy	Precision	Recall	F-score
XGBoost	0.9859	0.5837	0.7768	0.5837	0.6256
Decision tree	0.9775	0.6438	0.6613	0.6438	0.6487
SVM	0.9826	0.5	0.4913	0.5	0.4956
KNN	0.9812	0.5158	0.6159	0.5158	0.5321
Random forest	0.9848	0.5416	0.9962	0.5416	0.5732
Neural network	0.9826	0.5	0. 4913	0.5	0.4956

Table S21: weights of socio-economic features and climate features by using SHAP, RDC, and Pearson approach.

	SHAP		RDC		Pearson	
	socio-economic	Climate	socio-economic	Climate	socio-economic	Climate
Cluster1	0.6043	0.3957	0.4868	0.5132	0.1848	0.8151
Cluster2	0.5162	0.4838	0.4978	0.5022	0.3953	0.6046
Cluster3	0.6358	0.3642	0.4751	0.5248	0.4189	0.5810
Cluster4	0.2886	0.7114	0.5417	0.4583	0.7211	0.2788
Cluster5	0.2862	0.7138	0.5736	0.4264	0.6234	0.3765
Cluster6	0.3237	0.6763	0.5355	0.4645	0.6674	0.3325

CHIKV

For cluster 1:

Table S22: Prediction results of different machine learning approaches for cluster 1 in CHIKV clusters

Method	Accuracy	Weighted accuracy	Precision	Recall	F-score
XGBoost	0.9647	0.6632	0.8178	0.6632	0.7163
Decision tree	0.9639	0.7473	0.7772	0.7473	0.7636
SVM	0.9555	0.5	0.4777	0.5	0.4886
KNN	0.9494	0.5278	0.6065	0.5278	0.5434
Random forest	0.9632	0.6367	0.8465	0.6367	0.6921
Neural network	0.9578	0.5	0.4789	0.5	0.4893

For cluster 2:

Table S23: Prediction results of different machine learning approaches for cluster 2 in CHIKV clusters

Method	Accuracy	Weighted accuracy	Precision	Recall	F-score
XGBoost	0.9874	0.5589	0.8765	0.5589	0.5995
Decision tree	0.9833	0.6359	0.6265	0.6359	0.6308
SVM	0.9854	0.5	0.4943	0.5	0.4971
KNN	0.9867	0.5537	0.6331	0.5537	0.5757
Random forest	0.9874	0.5466	0.7595	0.5466	0.5732
Neural network	0.9884	0.5537	0.7013	0.5537	0.58

For cluster 3:

Table S24: Prediction results of different machine learning approaches for cluster 3 in CHIKV clusters

Method	Accuracy	Weighted accuracy	Precision	Recall	F-score
XGBoost	1.0	1.0	1.0	1.0	1.0
Decision tree	0.9473	0.5	0.4736	0.5	0.4864
SVM	0.9473	0.5	0.4736	0.5	0.4864
KNN	0.9473	0.5	0.4736	0.5	0.4864
Random forest	1.0	1.0	1.0	1.0	1.0
Neural network	0.9473	0.5	0.4736	0.5	0.4864

Table S25: weights of socio-economic features and climate features by using SHAP, RDC, and Pearson approaches.

	SHAP		RDC		Pearson	
	socio-economic	Climate	socio-economic	Climate	socio-economic	Climate
Cluster1	0.4272	0.5728	0.5189	0.4811	0.4458	0.5541
Cluster2	0.4296	0.5704	0.4879	0.5121	0.3970	0.6029
Cluster3	0.3621	0.6378	0.6103	0.3897	0.2629	0.7370

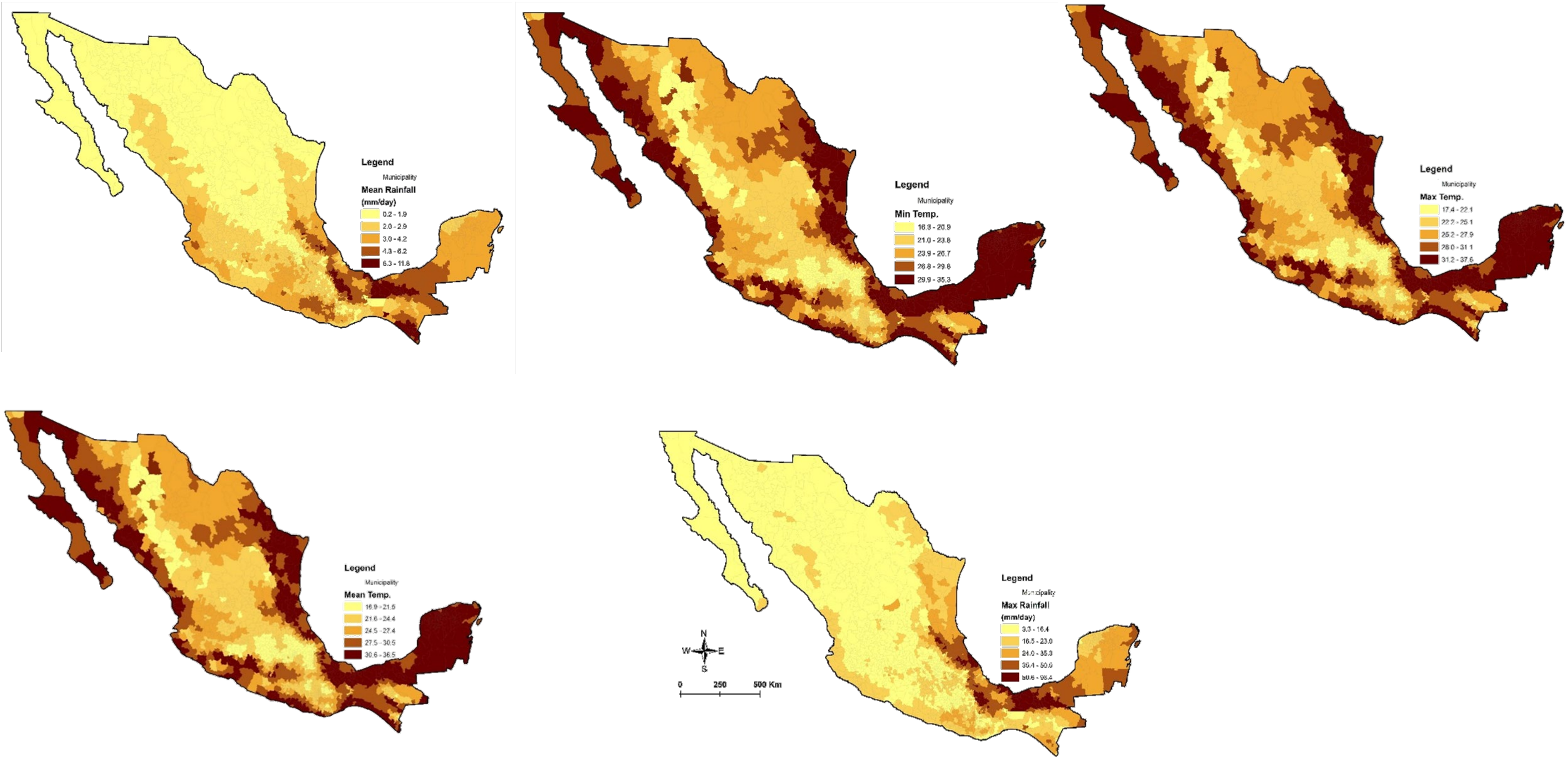

Figure S1: Spatial distribution of rainfall, and temperature in Mexico

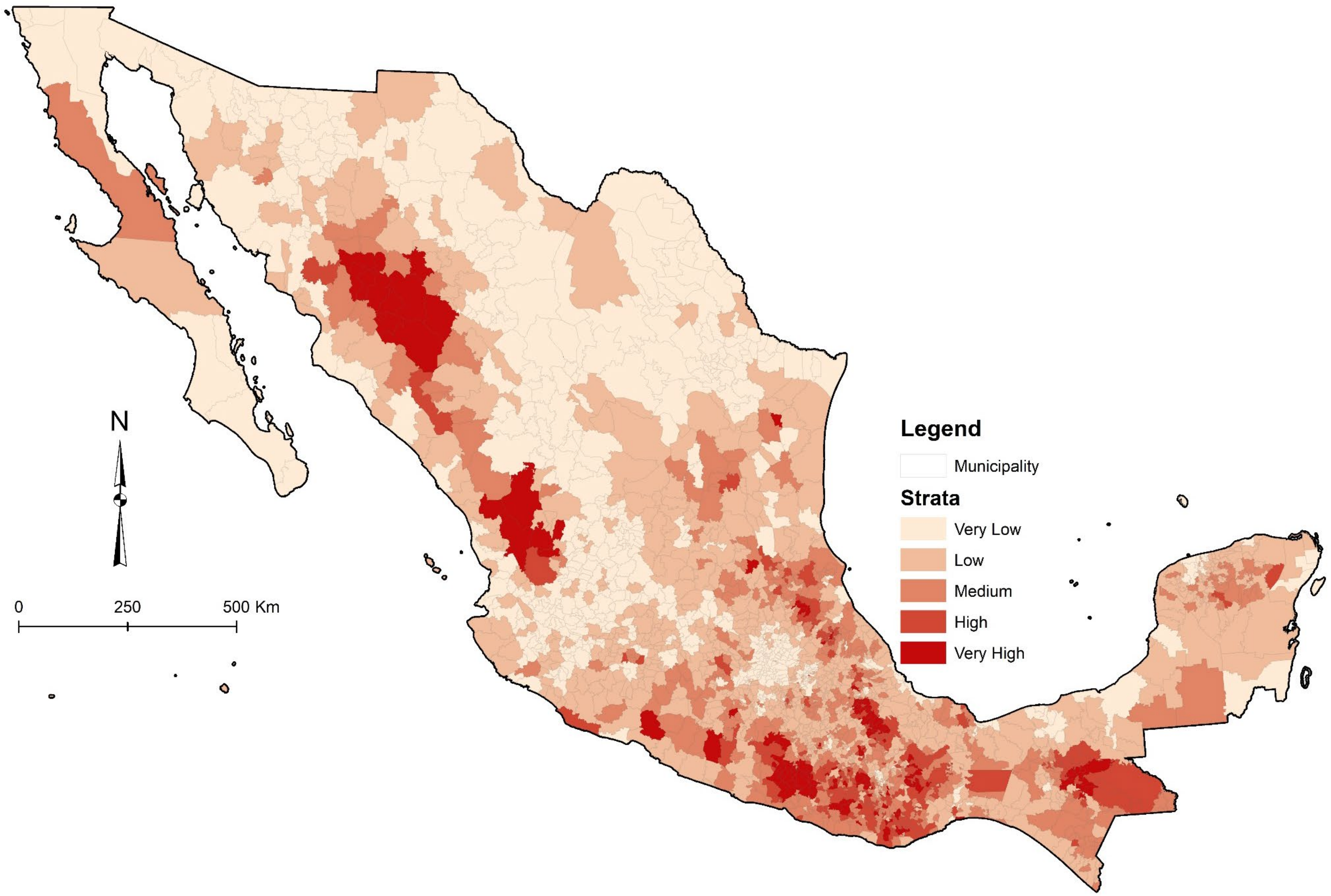

Figure S2: Socio-economic zones in Mexico

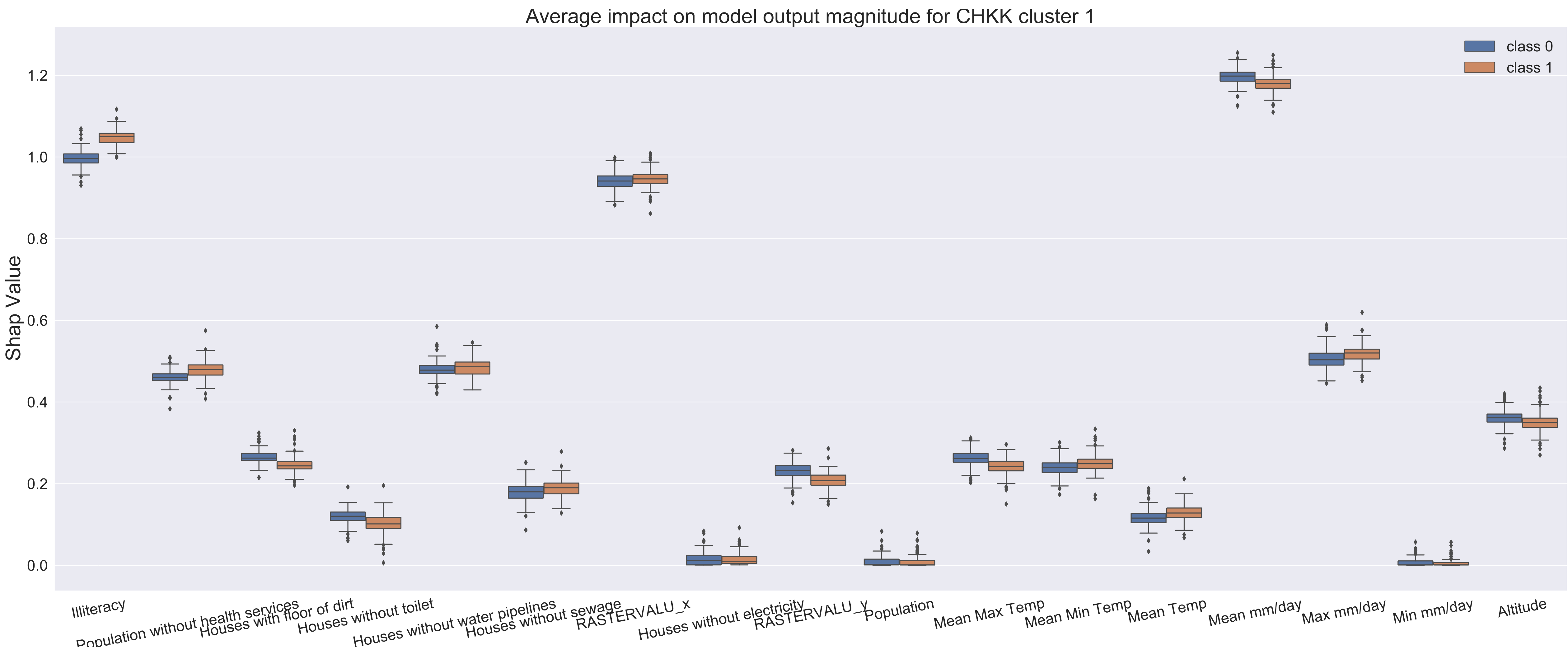

Figure S3: CHKK cluster 1

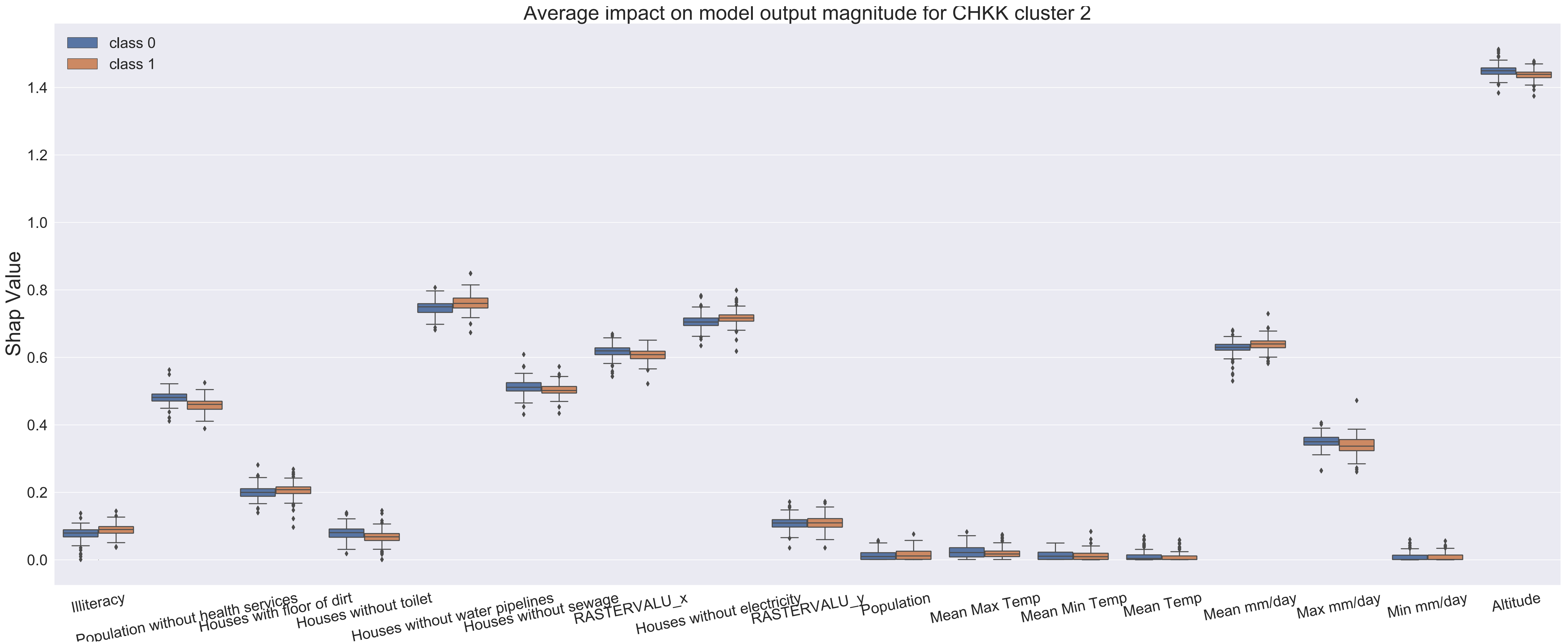

Figure S4: CHKK cluster 2

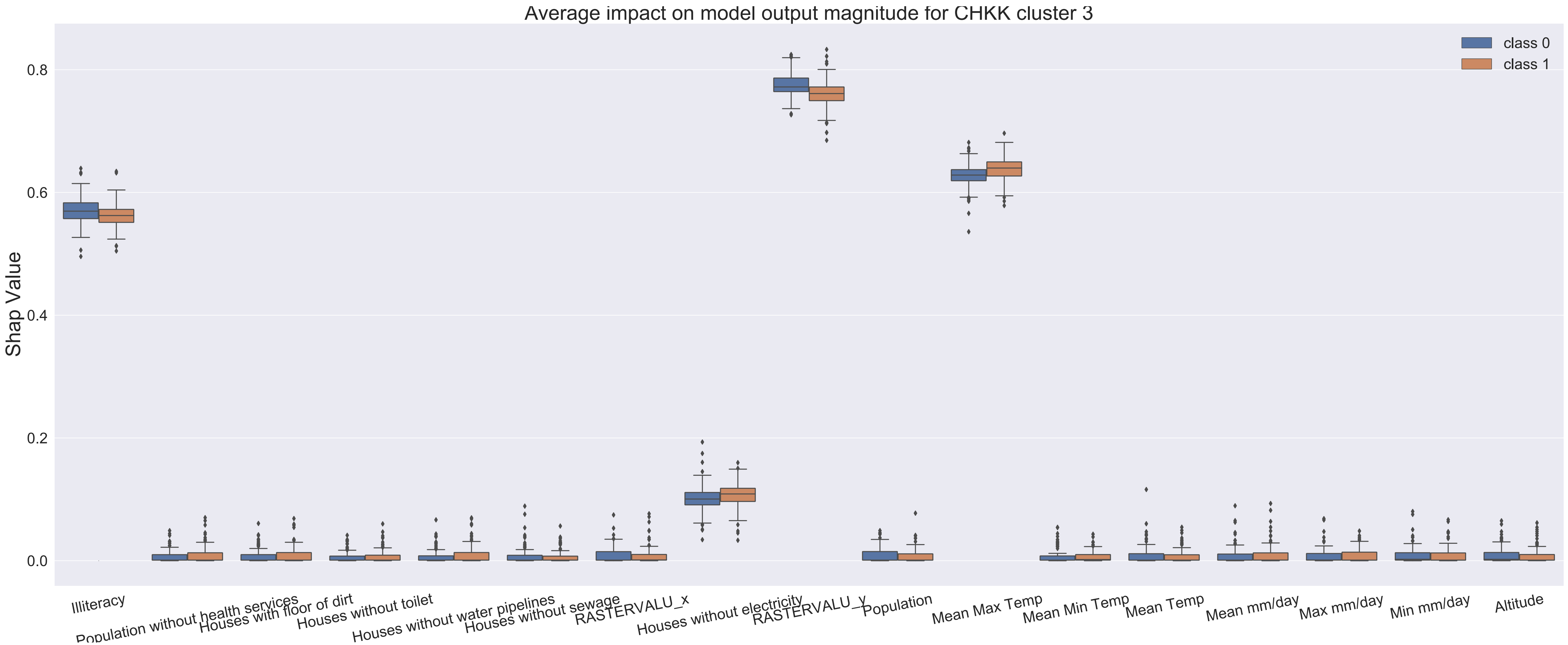

Figure S5: CHKK cluster 3

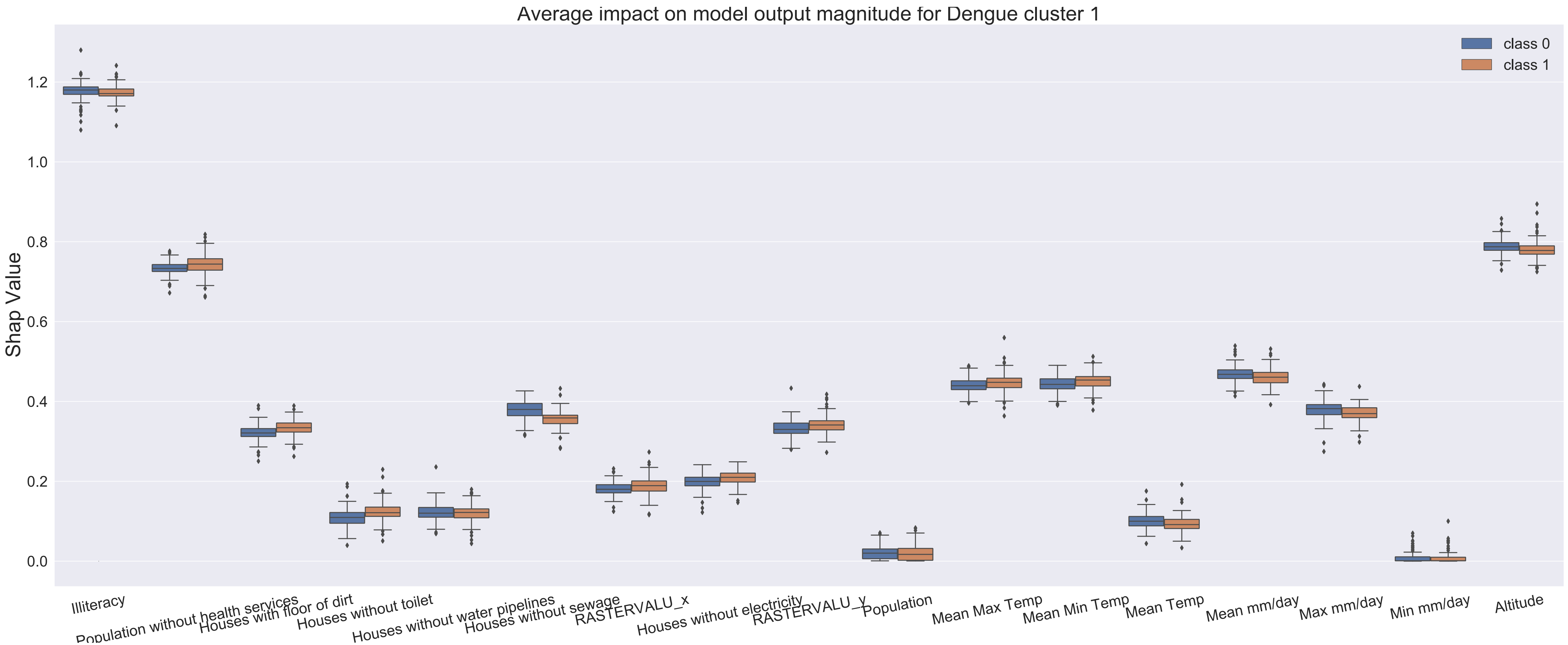

Figure S6: Dengue cluster 1

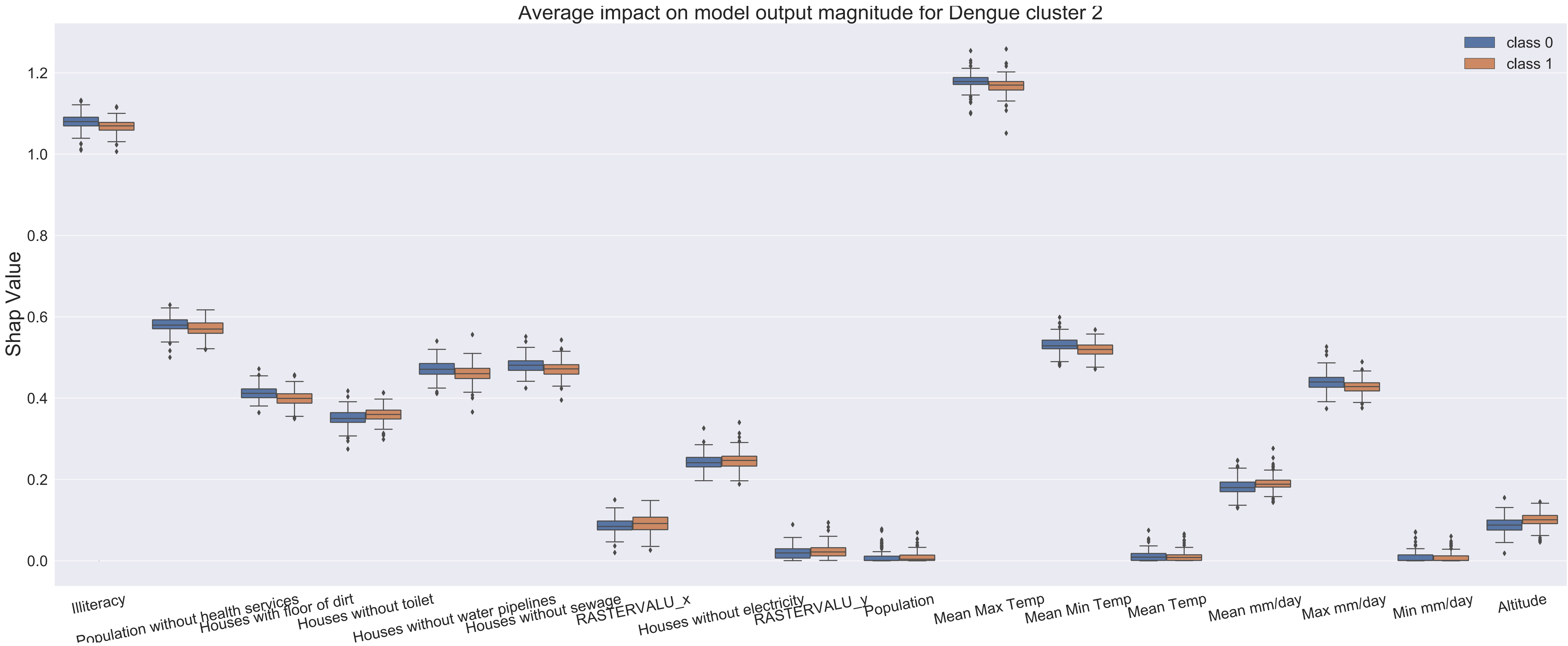

Figure S7: Dengue cluster 2

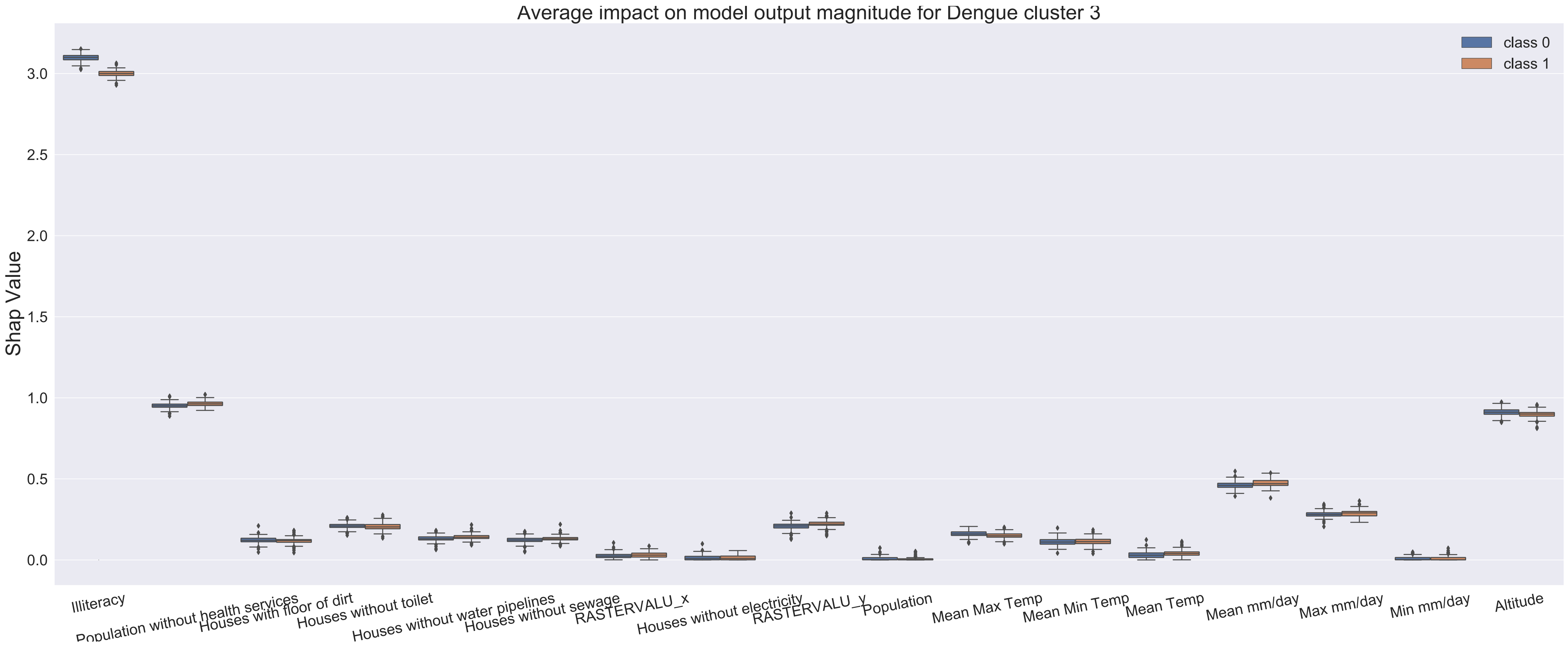

Figure S8: Dengue cluster 3

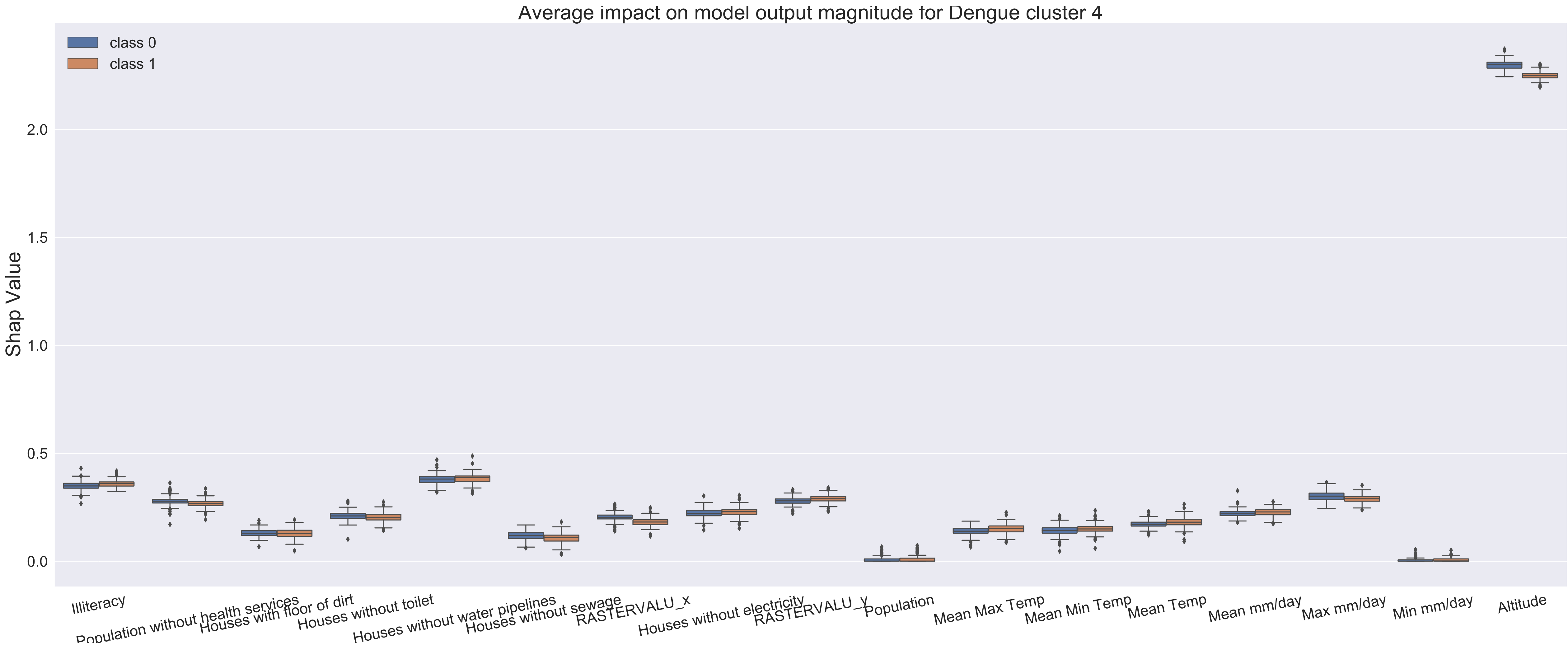

Figure S9: Dengue cluster 4

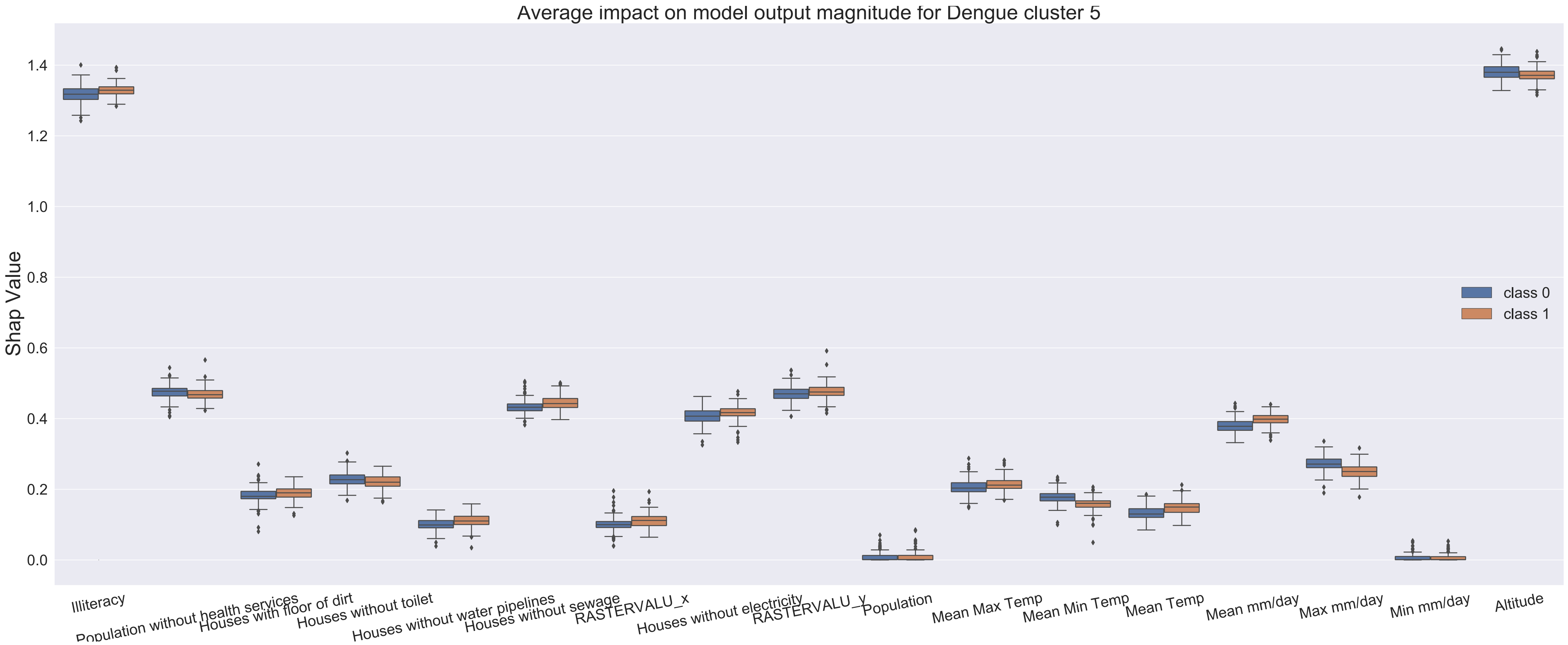

Figure S10: Dengue cluster 5

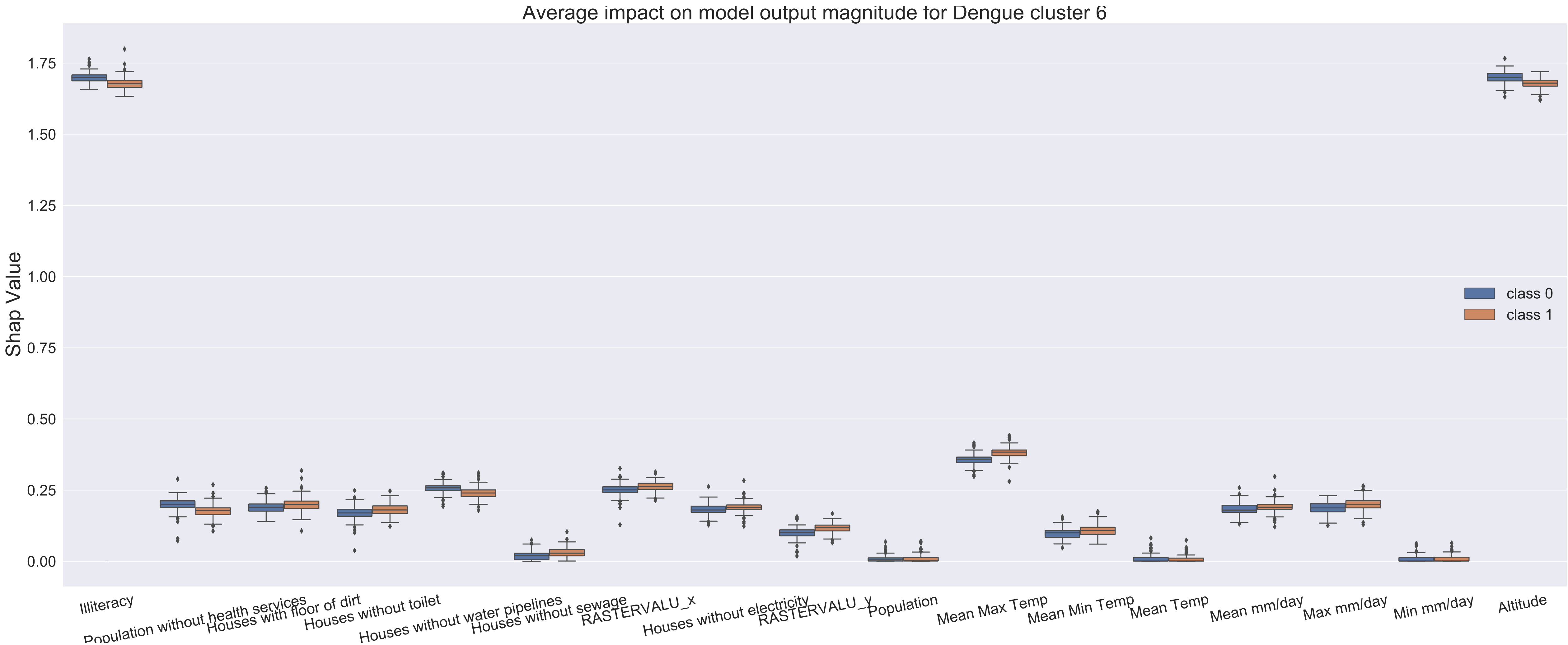

Figure S11: Dengue cluster 6

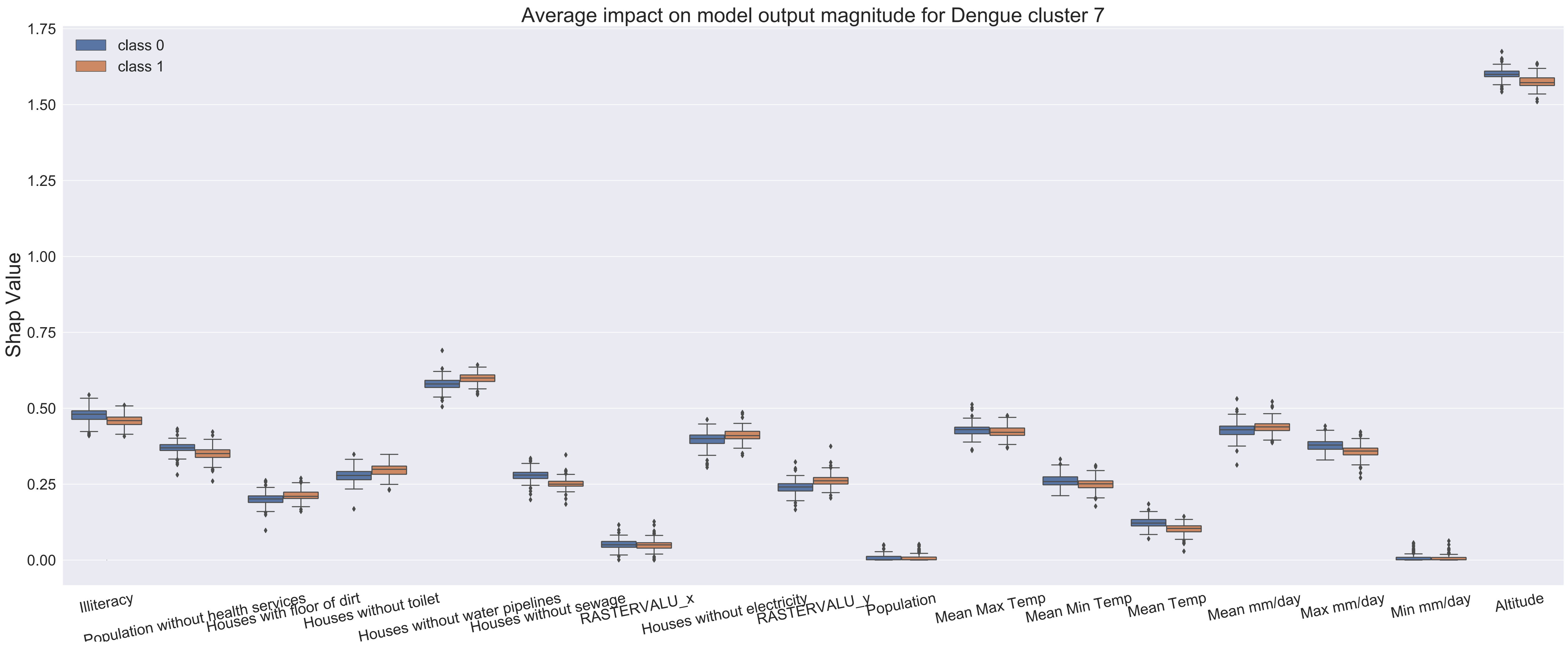

Figure S12: Dengue cluster 7

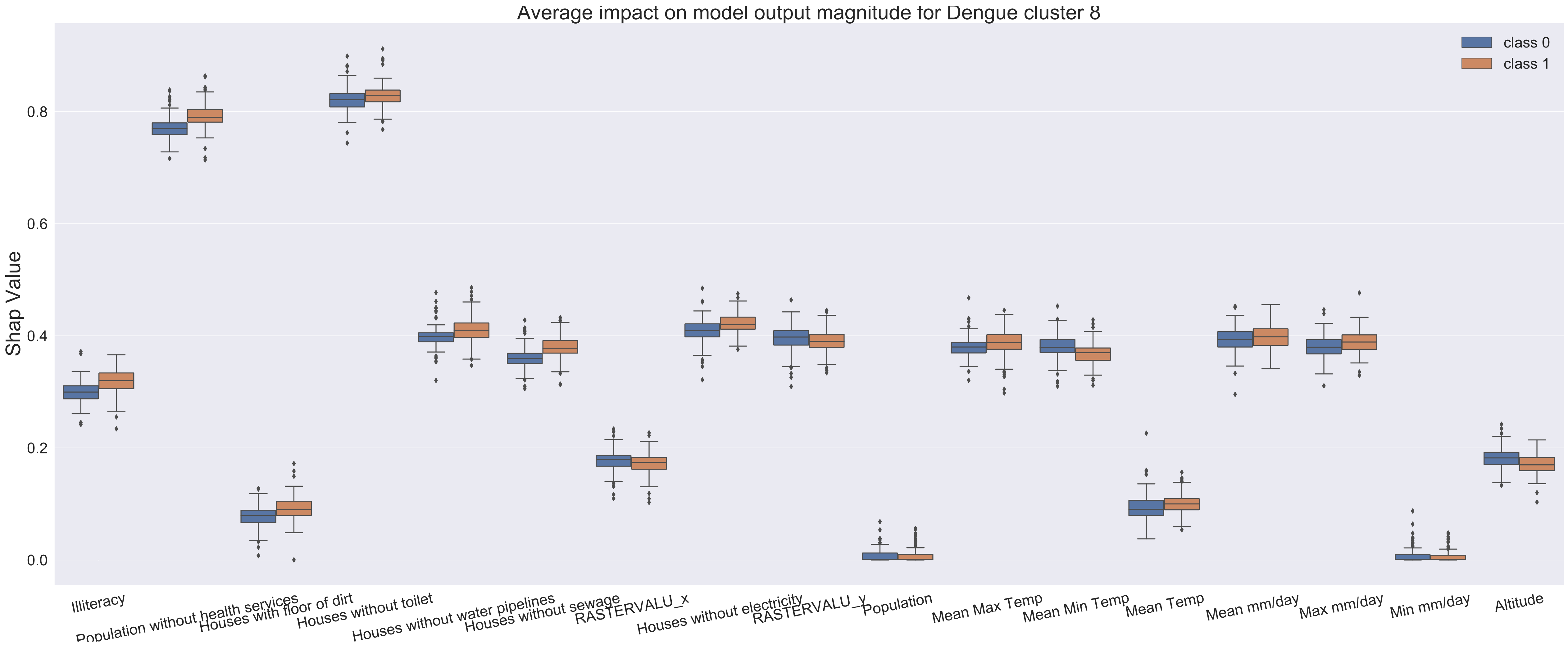

Figure S13: Dengue cluster 8

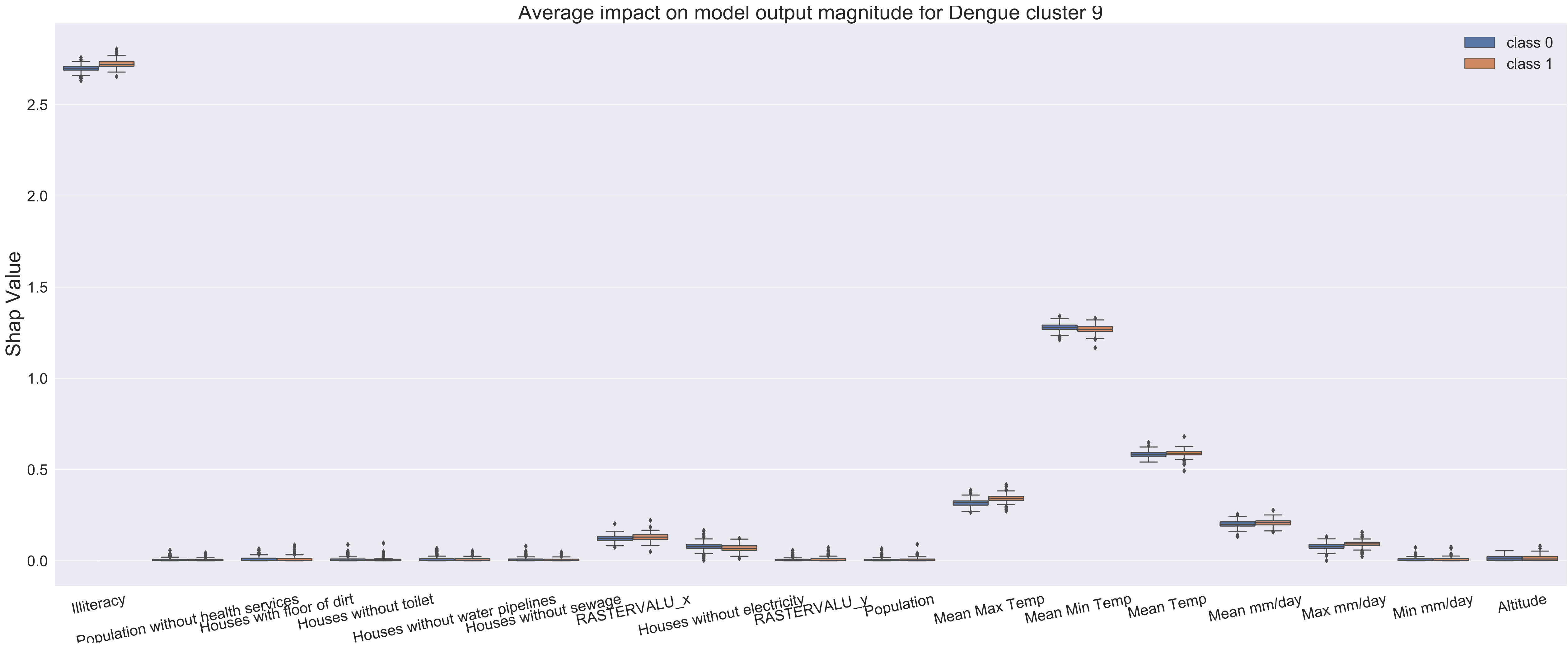

Figure S14: Dengue cluster 9

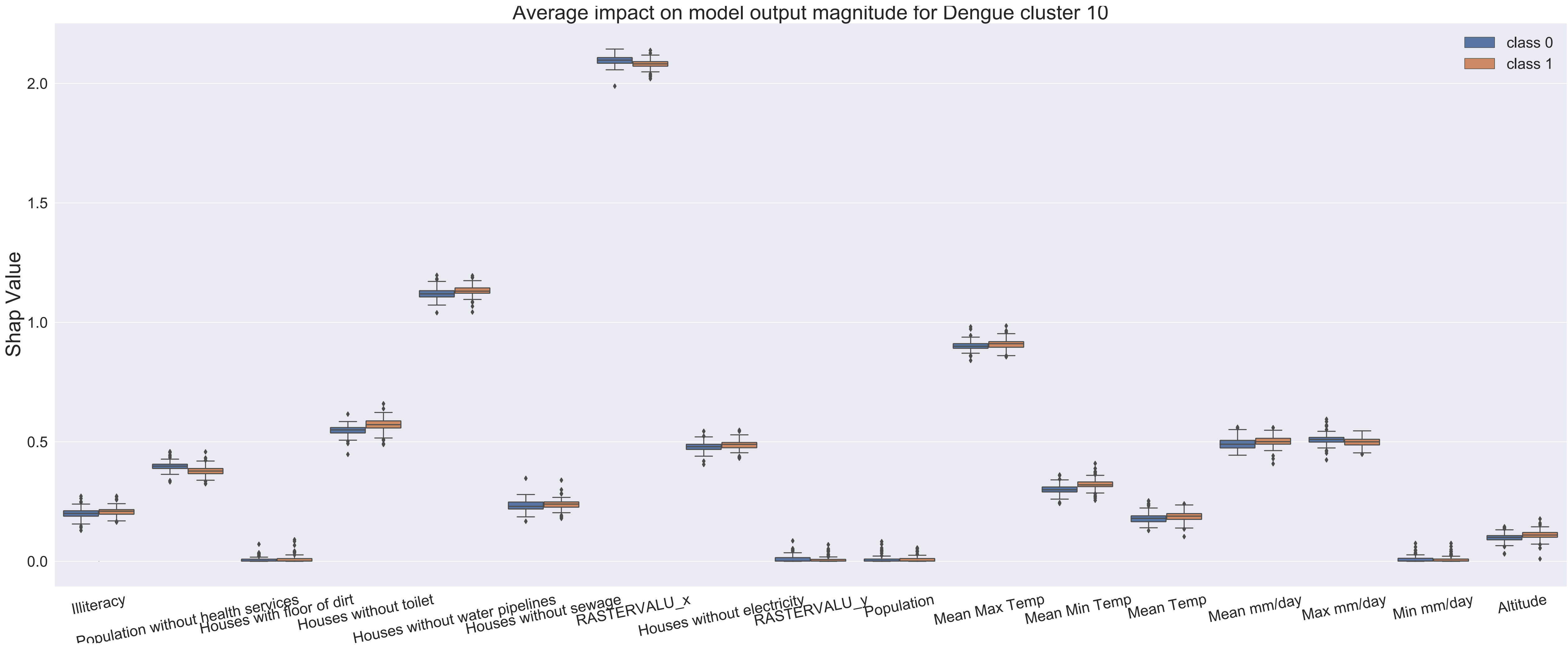

Figure S15: Dengue cluster 10

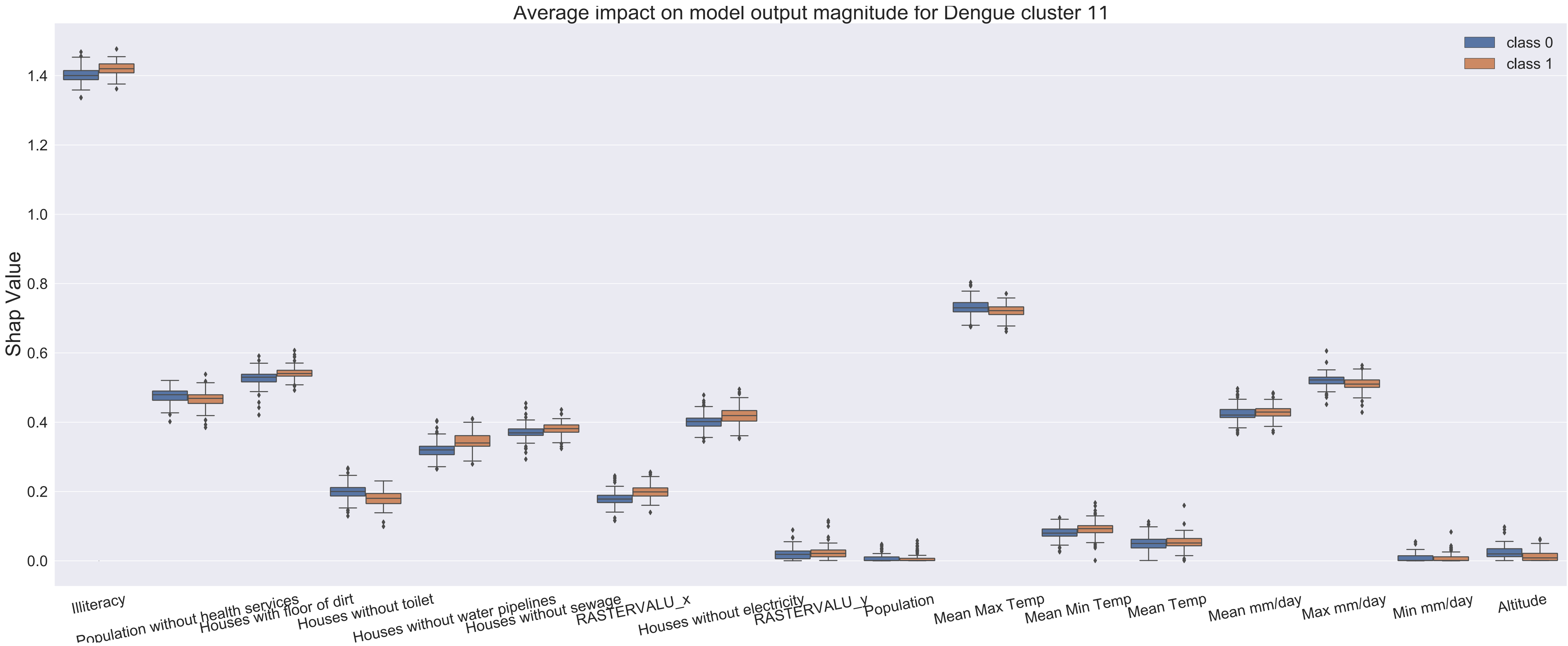

Figure S16: Dengue cluster 11

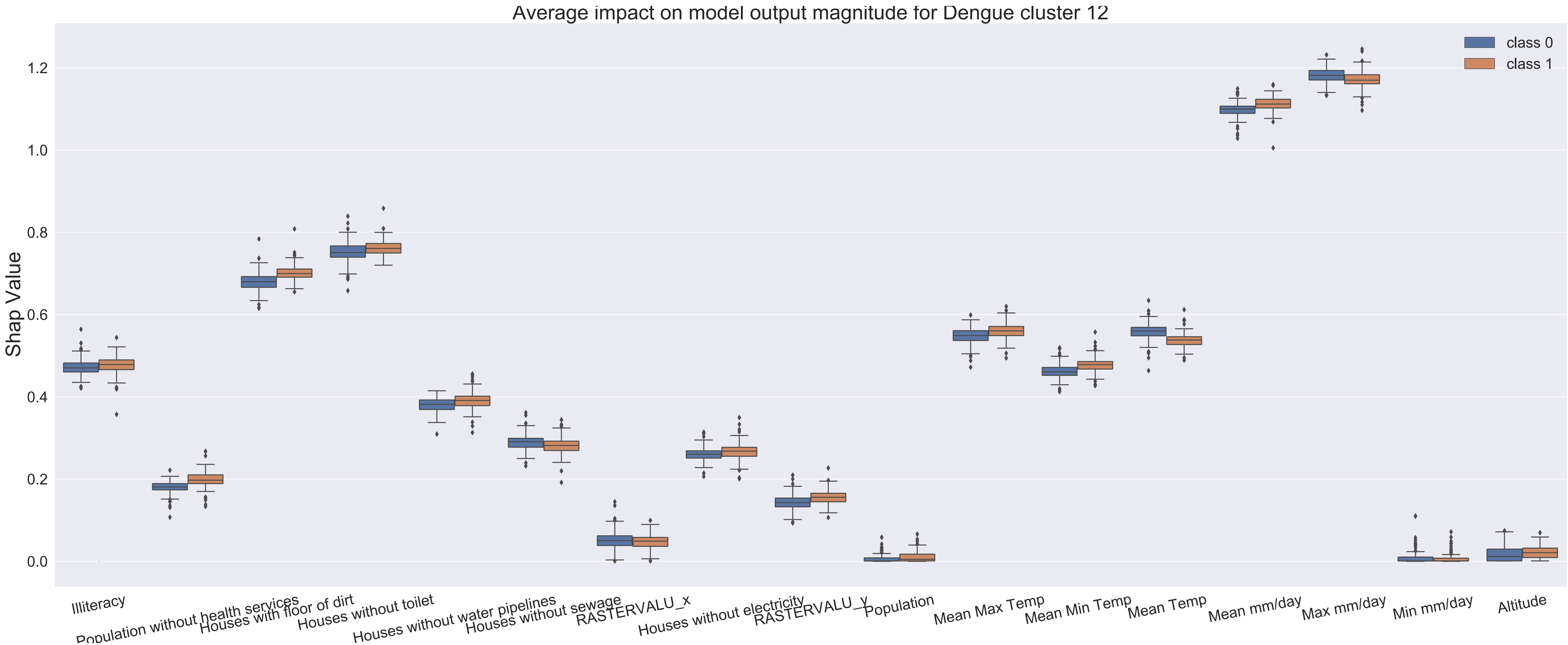

Figure S17: Dengue cluster 12

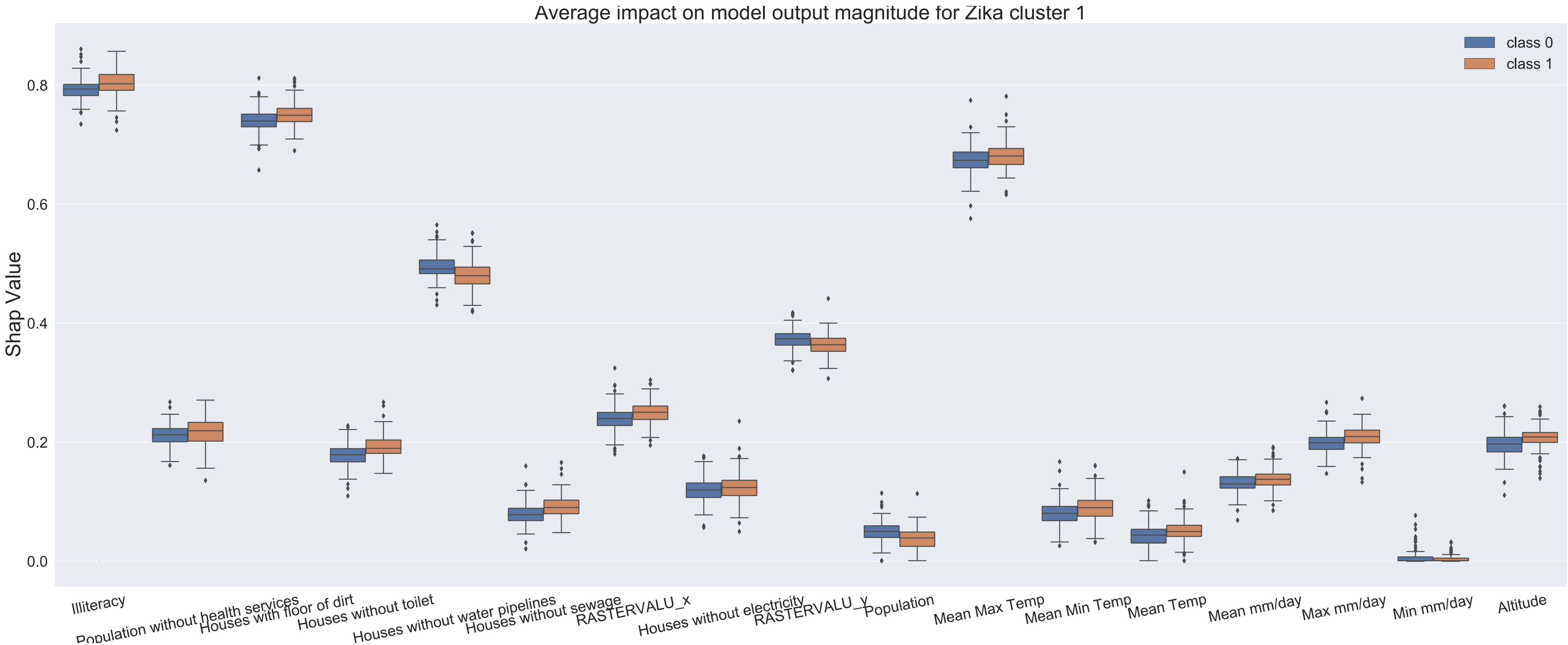

Figure S18: Zika cluster 1

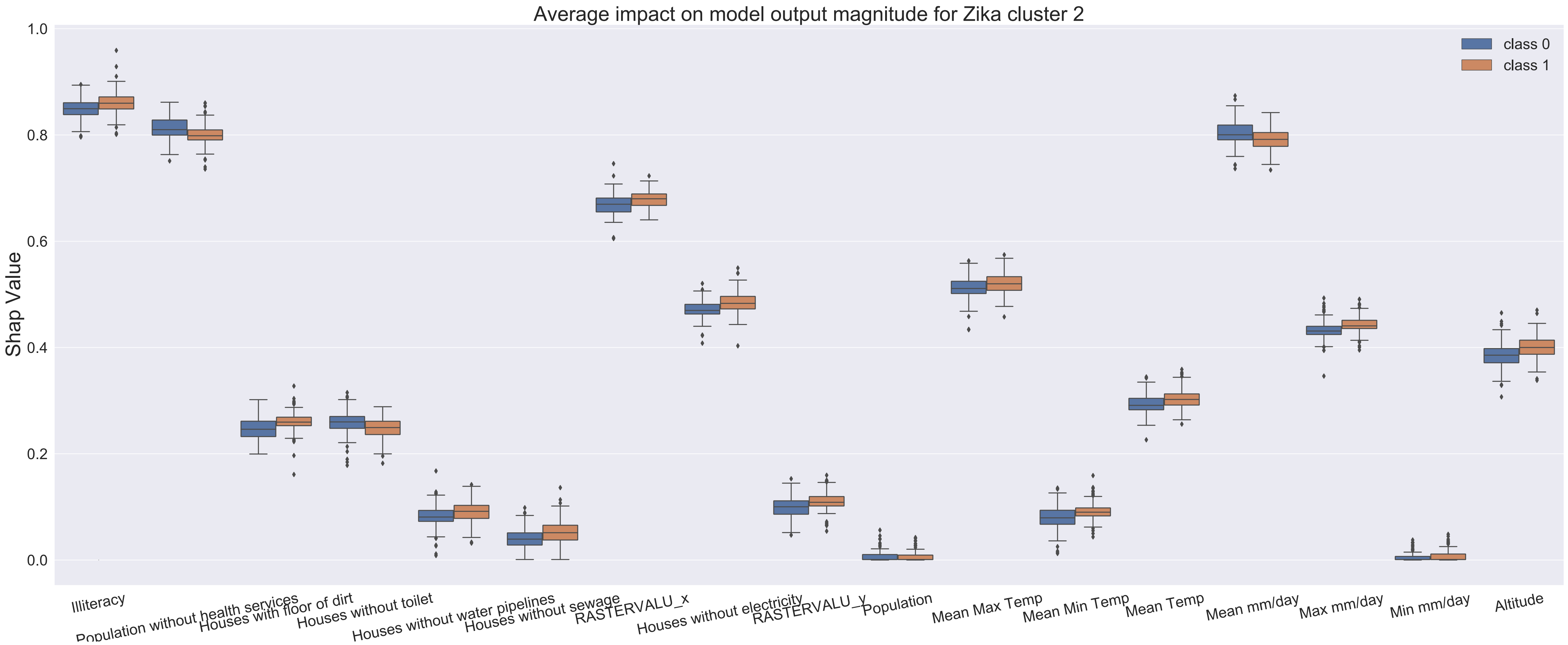

Figure S19: Zika cluster 2

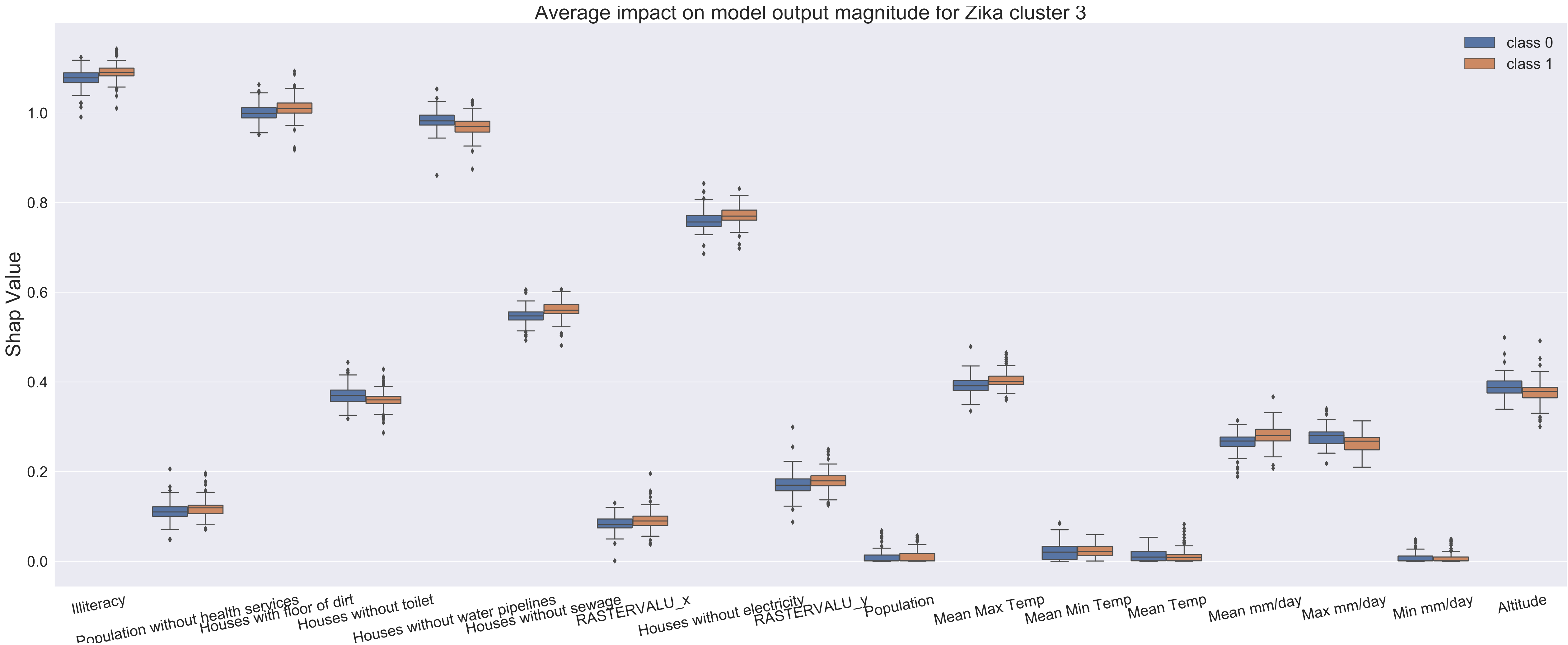

Figure S20: Zika cluster 3

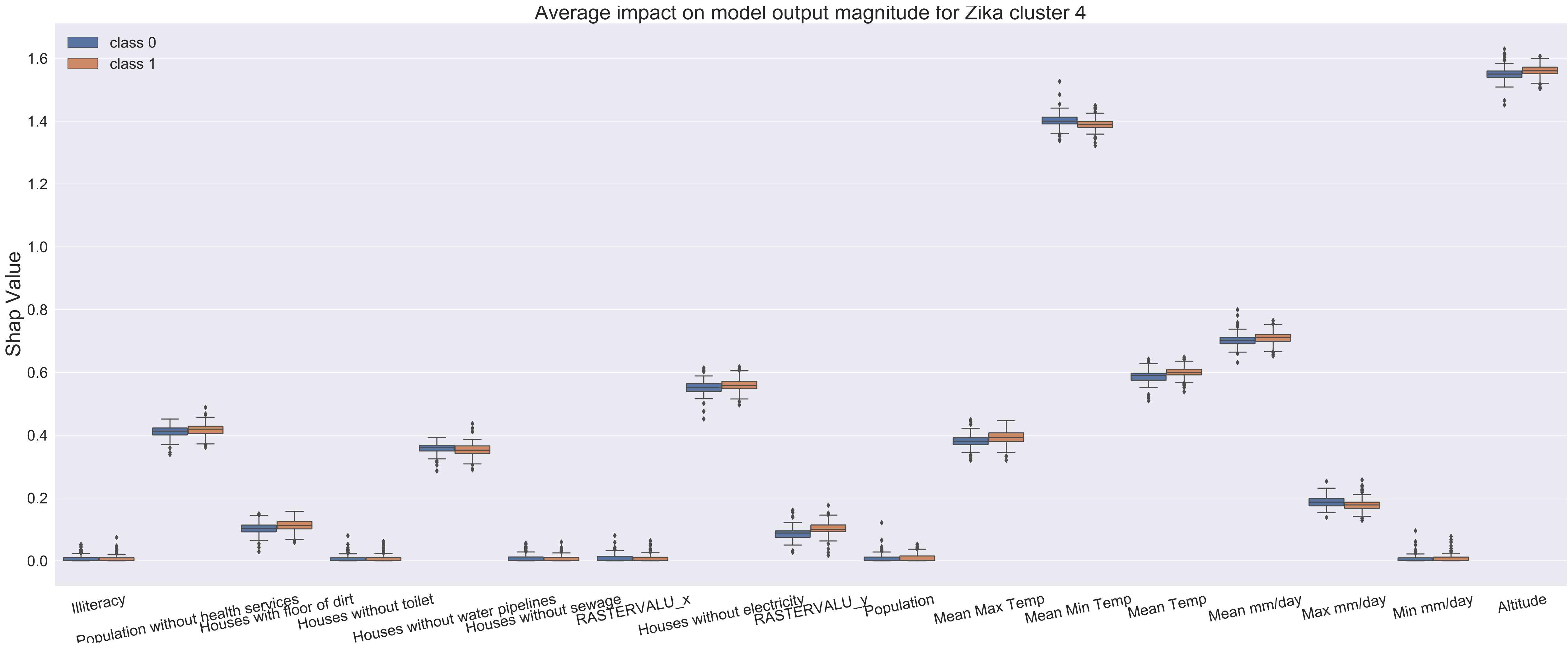

Figure S21: Zika cluster 4

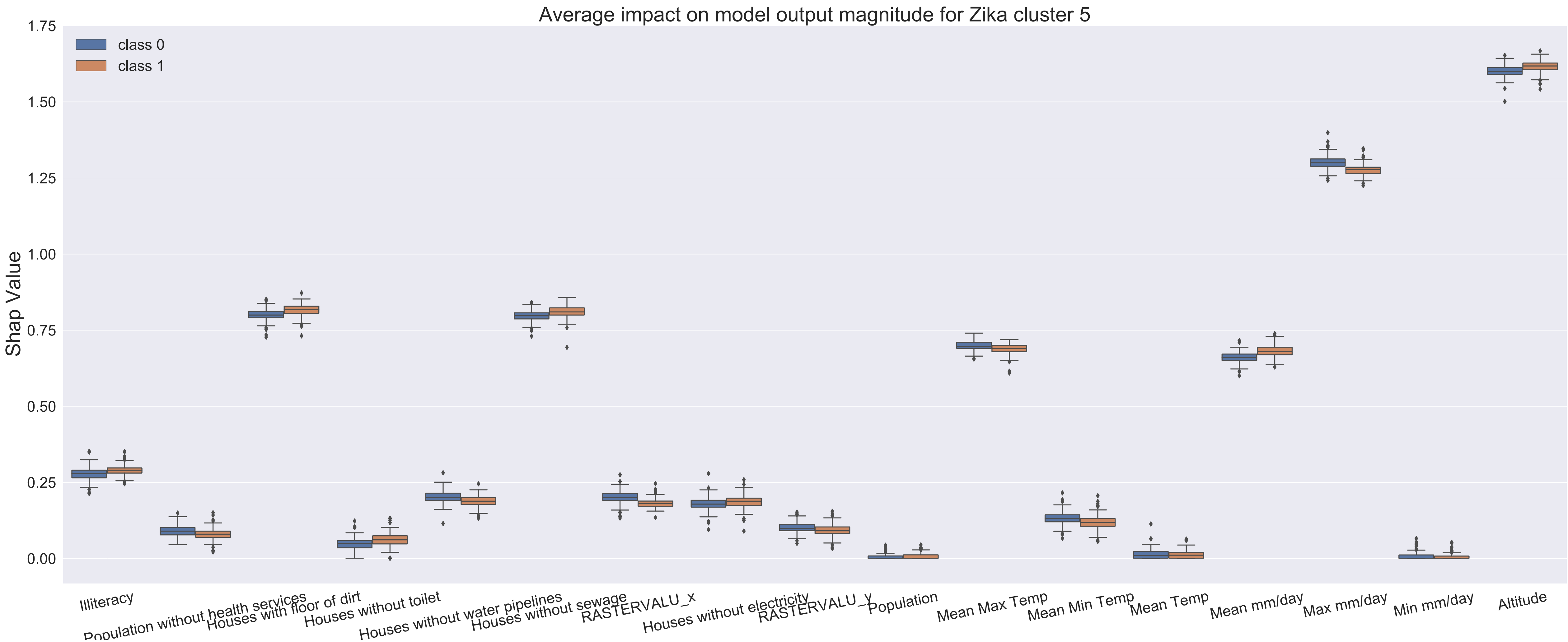

Figure S22: Zika cluster 5

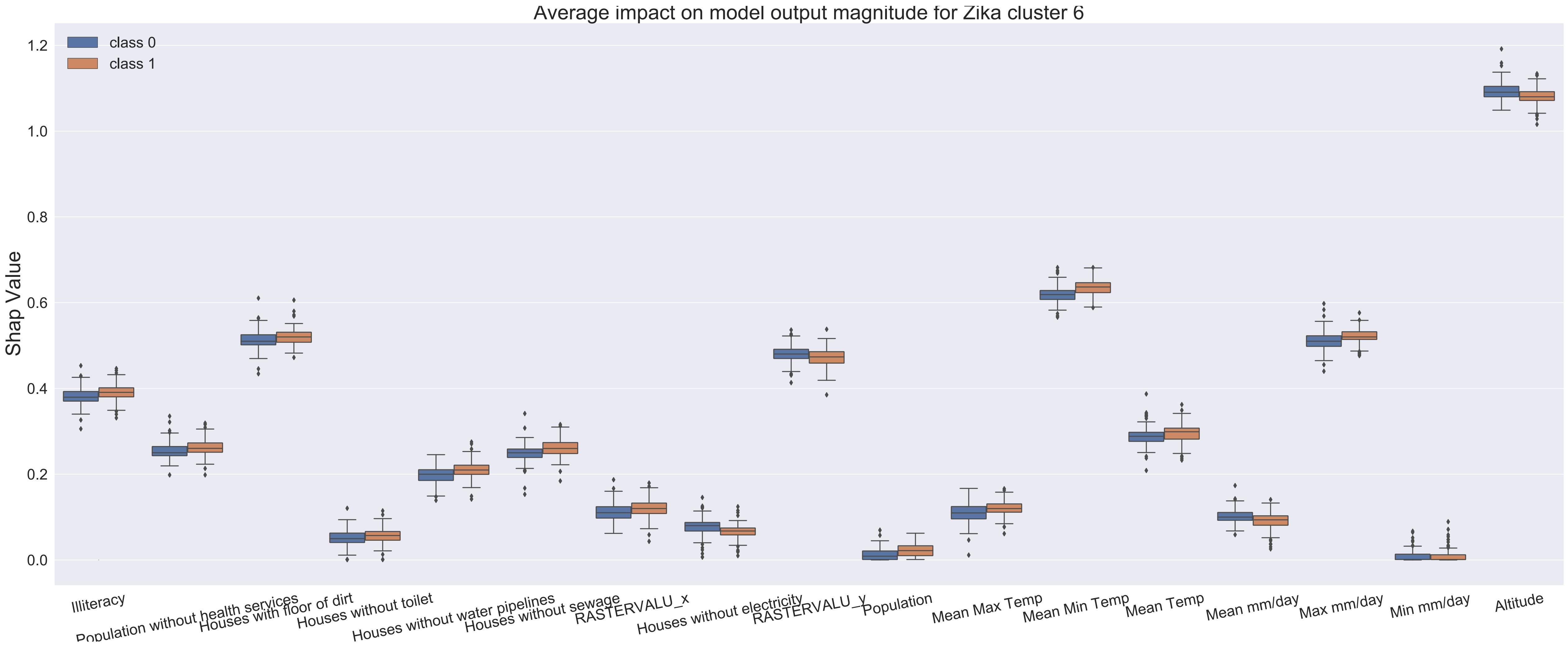

Figure S23: Zika cluster 6

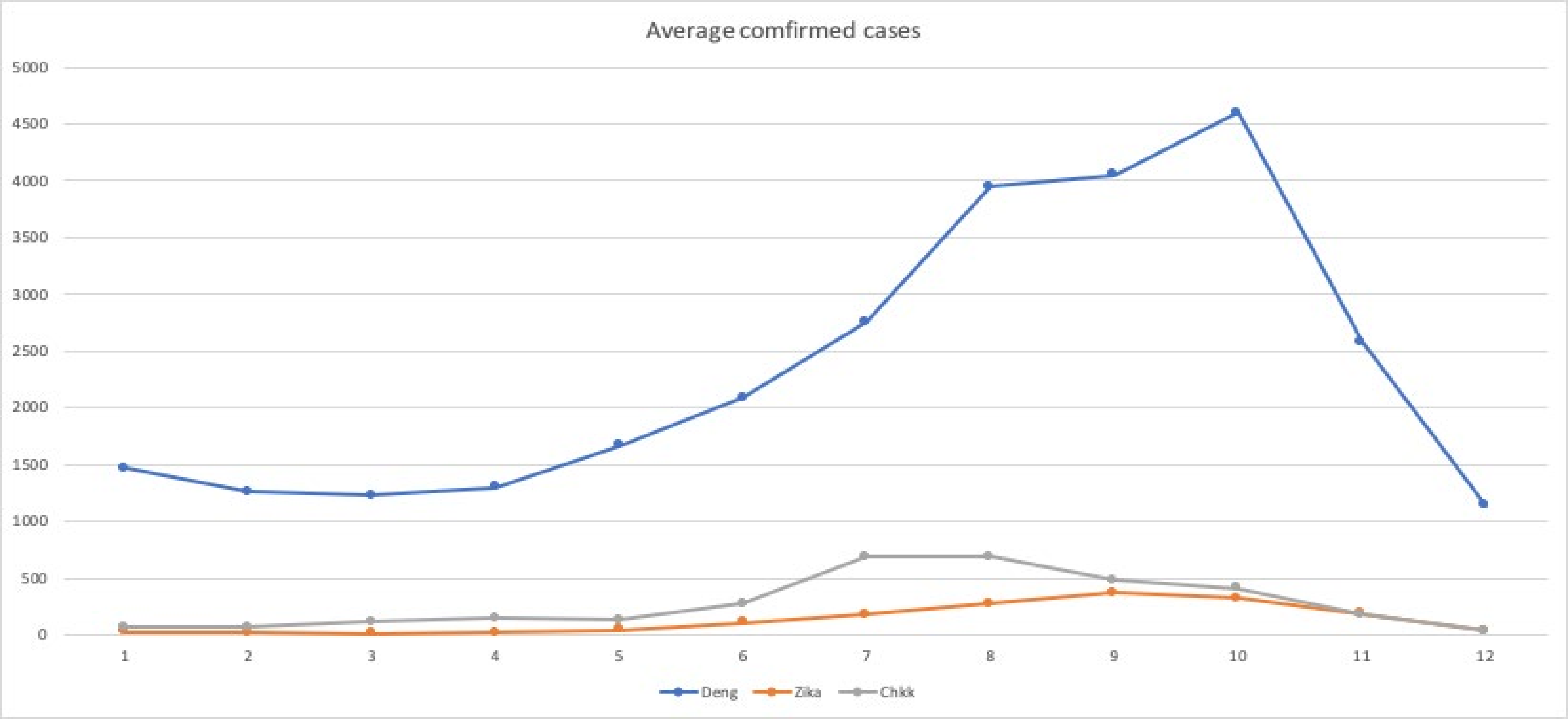

Figure S24: Average confirmed cases from Jan to Dec over 2012-2019
