# Supplementary material for: Spatio-temporal dynamics of three diseases caused by Aedes-borne arboviruses in Mexico
Source: Commun Med (Lond). 2022 Oct 28;2:134. doi: 10.1038/s43856-022-00192-7 (PMC9616936; doi:10.1038/s43856-022-00192-7)
Supplement: Supplementary file 2 — Description of Additional Supplementary Files [file 43856_2022_192_MOESM2_ESM.pdf]

Description of Additional Supplementary Files

File Name: Supplementary Data

Description: This is the model output dateset used for generating all main figures and tables
