# Supplementary material for: Spatio-temporal dynamics of three diseases caused by Aedes-borne arboviruses in Mexico
Source: Commun Med (Lond). 2022 Oct 28;2:134. doi: 10.1038/s43856-022-00192-7 (PMC9616936; doi:10.1038/s43856-022-00192-7)
Supplement: Supplementary file 5 — Reporting Summary [file 43856_2022_192_MOESM5_ESM.pdf]

nature research

Corresponding author(s): Ubydul Haque

Last updated by author(s): Sep 19, 2022

Reporting Summary

Nature Research wishes to improve the reproducibility of the work that we publish. This form provides structure for consistency and transparency in reporting. For further information on Nature Research policies, see our Editorial Policies and the Editorial Policy Checklist.

Statistics

For all statistical analyses, confirm that the following items are present in the figure legend, table legend, main text, or Methods section.

n/a	Confirmed	
☐	☒	The exact sample size (*n*) for each experimental group/condition, given as a discrete number and unit of measurement
☐	☒	A statement on whether measurements were taken from distinct samples or whether the same sample was measured repeatedly
☒	☐	The statistical test(s) used AND whether they are one- or two-sided<br>*Only common tests should be described solely by name; describe more complex techniques in the Methods section.*
☐	☒	A description of all covariates tested
☐	☒	A description of any assumptions or corrections, such as tests of normality and adjustment for multiple comparisons
☐	☒	A full description of the statistical parameters including central tendency (e.g. means) or other basic estimates (e.g. regression coefficient) AND variation (e.g. standard deviation) or associated estimates of uncertainty (e.g. confidence intervals)
☐	☒	For null hypothesis testing, the test statistic (e.g. *F*, *t*, *r*) with confidence intervals, effect sizes, degrees of freedom and *P* value noted<br>*Give P values as exact values whenever suitable.*
☒	☐	For Bayesian analysis, information on the choice of priors and Markov chain Monte Carlo settings
☒	☐	For hierarchical and complex designs, identification of the appropriate level for tests and full reporting of outcomes
☐	☒	Estimates of effect sizes (e.g. Cohen's *d*, Pearson's *r*), indicating how they were calculated

Our web collection on statistics for biologists contains articles on many of the points above.

Software and code

Policy information about availability of computer code

Data collection	No Software was used.
Data analysis	We used SaTScan (v. 9.6.1) to determine Clusters. We also used ArCGIS v10.7.1 for the creation of some maps.

For manuscripts utilizing custom algorithms or software that are central to the research but not yet described in published literature, software must be made available to editors and reviewers. We strongly encourage code deposition in a community repository (e.g. GitHub). See the Nature Research guidelines for submitting code & software for further information.

Data

Policy information about availability of data

All manuscripts must include a data availability statement. This statement should provide the following information, where applicable:

- Accession codes, unique identifiers, or web links for publicly available datasets
- A list of figures that have associated raw data
- A description of any restrictions on data availability

The arbovirus data (Chikungunya, dengue, and Zika virus) used in this study are not publicly downloadable but can be requested at their original sites. Parties interested in data access should visit the Mexican ministry of health website (https://www.gob.mx/salud/en, E-mail: petitionscitizens@salud.gob.mx). The source data for the figures are available in Supplementary Data (Excel).

Code to reproduce study findings is freely available and accessible at GitHub link: https://github.com/BoDong111/COMMSMED " and in zenodo submission (https://doi.org/10.5281/zenodo.7071115).

Field-specific reporting

Please select the one below that is the best fit for your research. If you are not sure, read the appropriate sections before making your selection.

☒ Life sciences ☐ Behavioural & social sciences ☐ Ecological, evolutionary & environmental sciences

For a reference copy of the document with all sections, see nature.com/documents/nr-reporting-summary-flat.pdf

Life sciences study design

All studies must disclose on these points even when the disclosure is negative.

Sample size	As this is a retrospective study and from routine surveillance data, all available data was used and no subsamples were collected.
Data exclusions	No data was excluded from the study
Replication	Cross-validation methods were used validate using training and testing sets
Randomization	Not Applicable to this study, as this is a analysis was done on all samples collected. Nonetheless, Randomized Dependence Coefficient and other methods were used to analyze the influence of independent factors on the dependent variables.
Blinding	Not applicable because the data is from routine collection and sample analysis is done by independent staff with no link to investigators

Reporting for specific materials, systems and methods

We require information from authors about some types of materials, experimental systems and methods used in many studies. Here, indicate whether each material, system or method listed is relevant to your study. If you are not sure if a list item applies to your research, read the appropriate section before selecting a response.

Materials & experimental systems

n/a	Involved in the study	
☒	☐	Antibodies
☒	☐	Eukaryotic cell lines
☒	☐	Palaeontology and archaeology
☒	☐	Animals and other organisms
☒	☐	Human research participants
☒	☐	Clinical data
☒	☐	Dual use research of concern

Methods

n/a	Involved in the study	
☒	☐	ChIP-seq
☒	☐	Flow cytometry
☒	☐	MRI-based neuroimaging
